# Research and Application of Tongue Image Segmentation Method Based on Lightweight Deep Learning

**DOI:** 10.3390/bioengineering13101201

**Published:** 2026-10-15

**Authors:** Yulin Gou, Ziyang Zhang, Haibei Song, Alan Wang

**Affiliations:** 1 School of Intelligent Medicine, Chengdu University of Traditional Chinese Medicine, Chengdu 611137, China; 17780496836@163.com (Y.G.); zzy867500@163.com (Z.Z.); 2 Auckland Bioengineering Institute, The University of Auckland, Auckland 1142, New Zealand; 3 Medical Imaging Research Center, Faculty of Medical and Health Sciences, The University of Auckland, Auckland 1023, New Zealand; 4 Centre for Co-Created Ageing Research, The University of Auckland, Auckland 1023, New Zealand; 5 Centre for Brain Research, The University of Auckland, Auckland 1023, New Zealand

**Keywords:** lightweight deep learning models, mobile device deployment, semantic segmentation, objective representation of traditional Chinese medicine, tongue diagnosis

## Abstract

Intelligent tongue diagnosis demands high-precision segmentation on resource-constrained mobile devices. All deep learning models outperform conventional approaches, improving mean intersection over union (mIoU) by approximately 9% and accelerating inference by more than two orders of magnitude. Among the evaluated models, UNet++ achieves the highest test mIoU (0.9543) and Dice (0.9764), with paired bootstrap analysis confirming statistically significant advantages over BiSeNet, PP-LiteSeg, and HRNet. BiSeNet achieves the fastest inference speed (310.03 FPS) and the highest mPA (0.9766), making it the most efficient model for real-time deployment. HRNet and PP-LiteSeg provide a favorable accuracy–efficiency balance, suitable for mobile-based tongue diagnosis. Deployment of PP-LiteSeg on three Android devices achieved inference latency of 233–262 ms and throughput of 3.2–3.4 FPS, demonstrating the feasibility of lightweight models on commodity mobile hardware. These findings establish an evidence-based framework for model selection in mobile tongue diagnosis, supporting AI deployment on portable devices for clinical assistance and remote monitoring.

## 1. Introduction

Tongue diagnosis represents one of the most mature and widely employed diagnostic modalities in Traditional Chinese Medicine (TCM). Nevertheless, conventional tongue diagnosis remains heavily dependent on physicians’ visual inspection and subjective experience, rendering it vulnerable to individual bias and environmental variations, including illumination conditions, imaging devices, and tongue protrusion posture [1,2]. Furthermore, the inefficient transmission of clinical expertise impedes large-scale clinical application [3,4,5]. A core limitation stems from the absence of standardized acquisition protocols and objective quantitative analytical methods, which has emerged as a major bottleneck in the modernization of tongue diagnosis [6,7].

**Figure 1:**
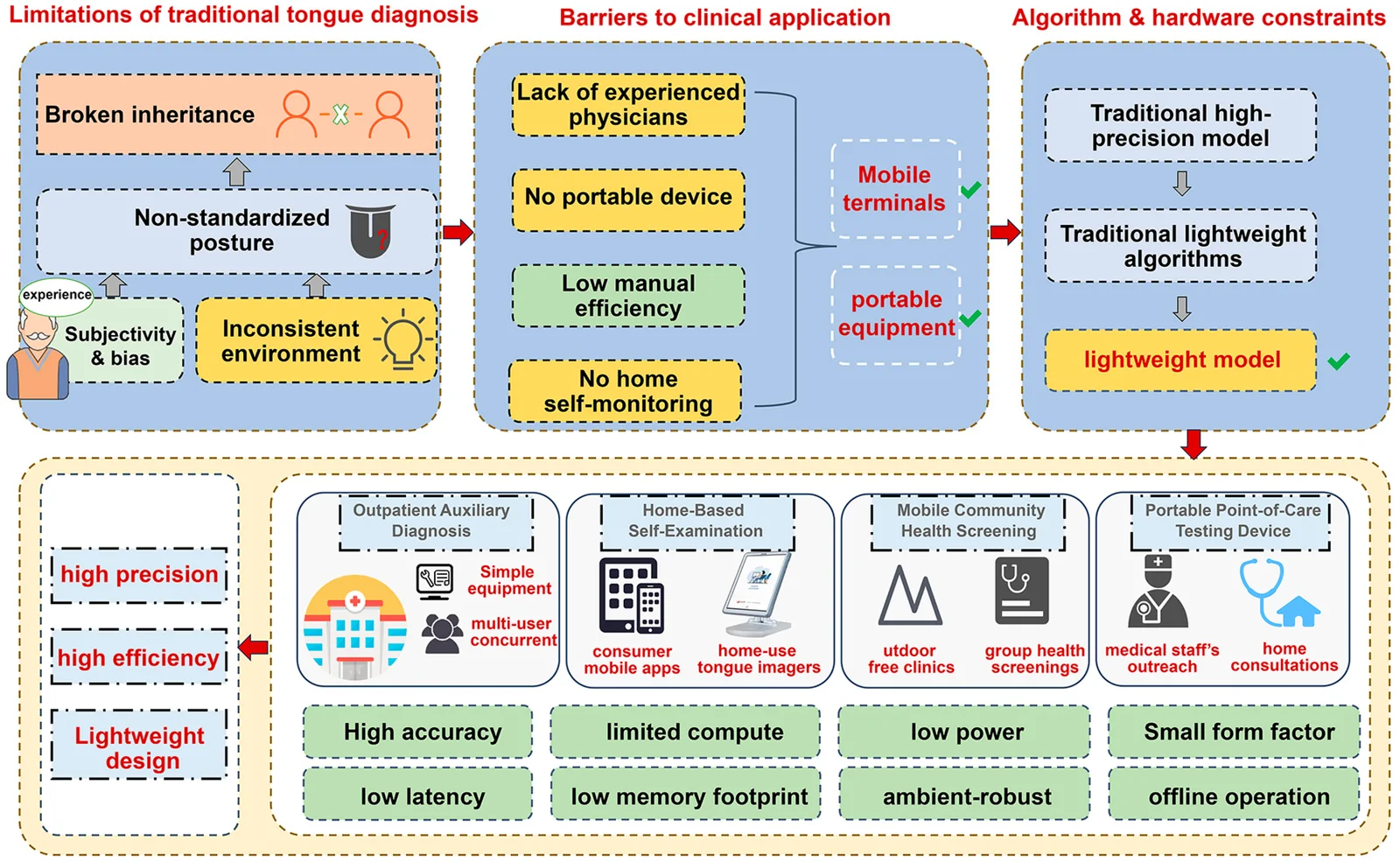
Schematic illustration of the rationale for lightweight deep learning in mobile traditional Chinese medicine (TCM) tongue diagnosis.

As illustrated in Figure 1, traditional tongue diagnosis encounters multiple barriers in clinical practice: a shortage of experienced practitioners in primary-level hospitals, insufficient low-cost portable equipment for community health screenings, and the lack of intelligent self-testing tools for home-based monitoring [8,9]. Concurrently, algorithmic and hardware constraints exacerbate these challenges: high-precision deep learning models typically entail considerable numbers of parameters and substantial computational overhead, rendering their deployment on smartphones or embedded devices infeasible. These conflicting demands collectively underscore the necessity of lightweight technical solutions. Transforming tongue diagnosis from a subjective, experience-dependent assessment into an objective, quantifiable technical system potentially deployable on resource-constrained edge devices has become a promising avenue for the modernization of tongue diagnosis [10].

Although prior studies have explored automated tongue diagnosis on mobile platforms, an inherent discrepancy persists between the computational requirements of high-precision models and the resource constraints of mobile devices. Existing research has predominantly focused on isolated algorithmic optimizations, whereas systematic investigation regarding model selection for resource-constrained deployment scenarios remains lacking. Consequently, insufficient empirical evidence is available to guide technology selection for practical implementation [11,12].

To address these gaps, this study systematically compares and selects lightweight tongue image segmentation models for mobile and portable devices. We construct a clinical tongue image dataset with pixel-level annotations and optimize conventional segmentation algorithms as baselines. Using a unified hardware platform and standardized input dimensions, we train and evaluate a comprehensive set of representative lightweight semantic segmentation models, encompassing mainstream technical paradigms, including encoder–decoder architectures, high-resolution representation preservation, dual-branch real-time designs, and lightweight transformer architectures. Model performance is comprehensively assessed through multidimensional metrics, encompassing segmentation accuracy, inference latency, and computational complexity. The ultimate objective is to identify suitable models for deployment in edge-based tongue diagnosis applications and portable diagnostic devices, thereby providing evidence-based model selection guidelines for primary care, community health screening, and home-based health monitoring.

## 2. Related Work

### 2.1. Tongue Image Segmentation: From Traditional Methods to Deep Learning

Several reviews have systematically summarized the progress in automated tongue diagnosis from different perspectives. Some studies reviewed the latest developments in automated tongue diagnosis technology and proposed a conceptual framework for mobile platforms [13]. Some scholars have comprehensively reviewed the application of artificial intelligence technology in tongue image calibration, detection, segmentation, and disease and syndrome classification in the past five years, emphasizing the research value of attention mechanisms, multi-scale features, and the combination of deep learning with traditional methods [14]. Additionally, some reviews have systematically summarized the digital analysis methods of tongue diagnosis, modern omics technologies, and their clinical applications, providing ideas for subsequent objective research on tongue diagnosis [15].

Tongue segmentation constitutes a fundamental step in precisely isolating the tongue region from the original image and represents the initial stage in objectifying tongue diagnosis [16]. Accurate separation of the tongue from the background (e.g., lips, skin, and teeth) is essential for subsequent tasks, including tongue color analysis, coating classification, and detection of cracks and tooth marks, to be performed on reliable data. However, the segmentation task faces multiple challenges: tongue edges exhibit color similarity to the lips, with boundaries becoming blurred under uneven illumination; cracks, tooth marks, and tongue coatings of varying thickness interfere with region identification; and variations in clinical imaging equipment and environmental conditions further increase uncertainty. Early conventional image segmentation methods, such as the Snake active contour model [17] and the GrabCut graph-cut algorithm [18], can accomplish basic region extraction; however, they remain sensitive to initial contour placement, rely on assumptions regarding color distribution, exhibit limited robustness, and require several seconds to tens of seconds per image, thereby failing to satisfy real-time constraints of portable devices [19,20].

Classic deep learning models such as U-Net [21] and the DeepLab series [22] achieved high accuracy on graphics processing units (GPUs), but entail substantial parameter counts, restricting their deployment to high-performance PCs or servers and precluding direct application in resource-constrained scenarios such as mobile phones and embedded devices. To solve these problems, researchers designed specialized lightweight architectures: BiSeNet adopts a dual-branch structure to balance details and speed [23]; PP-LiteSeg achieves the optimal balance of accuracy and efficiency through a unified attention fusion module [24]; SegNeXt-T uses multi-scale convolutional attention to simulate attention in a pure convolutional manner, avoiding the high computational cost of Transformers [25]; SegFormer uses a hierarchical Transformer without position encoding and a lightweight multilayer perceptron (MLP) decoder [26].

In the tongue image segmentation task, Huang et al. improved U-Net by introducing residual structures and visual saliency detection [27]; Zhang et al. strengthened the DeepLabV3 decoder [28]; Wen et al. improved the tongue edge fine structure by using HRNet [29]; Tan et al. proposed Tongue-LiteSAM for lightweight distillation and zero-shot segmentation [30]; Wang et al. designed a lightweight model with parallel self-attention and progressive upsampling [31]. Peng et al. proposed CRANet, which demonstrated competitive performance in the joint task of tongue image segmentation and classification [32]; Ma et al. further improved the edge detail retention ability of the tongue image segmentation algorithm based on high-resolution features [33]. In recent years, the design ideas of lightweight architectures have become more diversified. Gao et al. proposed the U-MLP network, which is based on the MLP architecture and uses ReMLP blocks. Compared with convolutional neural networks (CNNs) and Transformers, it has smaller computational cost and fewer parameters, achieving Dice scores of 88.27%, 97.61%, and 95.91% in skin lesion, spleen, and left atrium segmentation tasks [34]. Alahmadi proposed a dual-path parallel encoder model that captures local semantics through the CNN branch and extracts global context through the Transformer branch, and then adaptively fuses them through a convolutional long short-term memory (ConvLSTM), effectively balancing local details and long-range dependencies [35]. Liu et al. proposed a hybrid model that uses pyramid convolution modules and multi-scale large kernel attention, achieving excellent performance on small-scale medical datasets [36]. Additionally, Wang et al. proposed O-Net, which achieves simultaneous optimization of segmentation and classification by deeply integrating CNN and Transformer [37]. For efficient learning, Zhang et al. proposed the Learned Mumford-Shah Network (LMS-Net), which combines the Mumford-Shah variational model with a deep unfolded network. By alternating optimization of prototype and mask updates, it enables few-sample medical image segmentation and offers a high-interpretability approach for tongue image segmentation in data-scarce scenarios [38]. Wu et al. proposed the Collaborative Teaching and Representation Collaborative Learning (CTRCL) framework, which enables bidirectional knowledge transfer between CNN and Transformer models through logit collaborative learning and category-aware feature collaborative learning, achieving the best results on multiple medical segmentation benchmarks [39]. Liu et al. proposed Light-UNETR, which employs a lightweight dimension-reduction attention module and a context-collaborative enhanced learning strategy. With only 10% labeled data, the Jaccard index of left atrium segmentation improved by 1.43%, while floating-point operations (FLOPs) were reduced by 90.8% and the parameter count was reduced by 85.8% [40]. In the direction of extreme lightweighting, Juraev et al. proposed RepSegNet, which designs the RepMobileUnit module using structural reparameterization. During training, multi-branch structures enhance feature representation; during inference, these are reparameterized into a single branch to significantly reduce computational costs, enabling efficient mobile deployment on medical segmentation datasets such as the International Skin Imaging Collaboration (ISIC) 2017 dataset and Kvasir-SEG [41]. Mini-Net compresses the network parameter quantity to only 37,800, maintaining extremely low computational costs while achieving state-of-the-art (SOTA) performance on multiple medical image benchmarks, providing new technical possibilities for resource-limited embedded tongue diagnosis devices [42]. However, most of these studies were validated on disparate datasets and hardware platforms, precluding systematic horizontal comparisons of multiple lightweight models on the same platform and the same evaluation system, resulting in the lack of objective and quantifiable empirical evidence for technology selection. In recent years, models represented by the Segment Anything Model (SAM) have achieved breakthroughs in general segmentation, but their parameter quantities are huge and cannot be directly deployed on mobile devices. Therefore, MobileSAM compresses SAM to a lightweight CNN backbone through knowledge distillation; EdgeSAM further optimizes edge device inference [43]; Zhong et al. proposed a zero-shot multimodal tongue body segmentation method based on SAM, further expanding the application of lightweight large models in the field of tongue diagnosis [44]; TongueSAM_Lite achieved a 42.7% reduction in model size through knowledge distillation [45]; MEAT-SAM optimized the automatic prompt generation mechanism [46]. For the problem of class imbalance in tongue image data, Lee et al. proposed a soft-labeling strategy based on Mosaic data augmentation and curriculum learning, effectively improving the generalization performance of tongue lesion classification models on imbalanced datasets [47]. Wu et al. introduced TransUNet to the tongue coating segmentation task, using the Transformer encoder to capture global context information, effectively addressing the limitations of traditional CNNs in segmenting tongue coatings of uneven thickness [48]. These works demonstrate the potential of lightweighting large models, but they are still in the stage of technical exploration, lack empirical validation on clinical tongue image datasets, and have not undergone fair comparison with mainstream lightweight CNN/Transformer models, limiting their maturity for practical application [49].

### 2.2. Lightweight Segmentation Models

The core feature of lightweighting techniques lies in significantly reducing the number of model parameters and computational load through a series of synergistic optimization methods, while minimizing any loss in model accuracy. This facilitates rapid inference and high energy efficiency, thereby enabling efficient deployment on resource-constrained platforms such as smartphones and embedded devices [50]. In recent years, numerous lightweight solutions have emerged in the field of deep learning-based medical image segmentation, spanning the complete technical spectrum from efficient backbone design to model compression. Specifically, these techniques are primarily implemented at three levels: first, designing efficient network architectures (e.g., deep separable convolutions, batch convolutions, and lightweight Transformers) to reduce computational load fundamentally; second, applying model compression techniques (e.g., knowledge distillation, pruning, and quantization) to pre-trained large models; and third, adopting collaborative optimization strategies (e.g., mixed-precision training and efficient decoders) during both training and inference phases to enhance operational efficiency further. A systematic review has summarized the latest advances in this field, discussed computational cost challenges in detail, and analyzed the design principles of representative lightweight models such as CoTr and ICGNet, providing a theoretical reference for model selection in resource-constrained scenarios [51].

**Figure 2:**
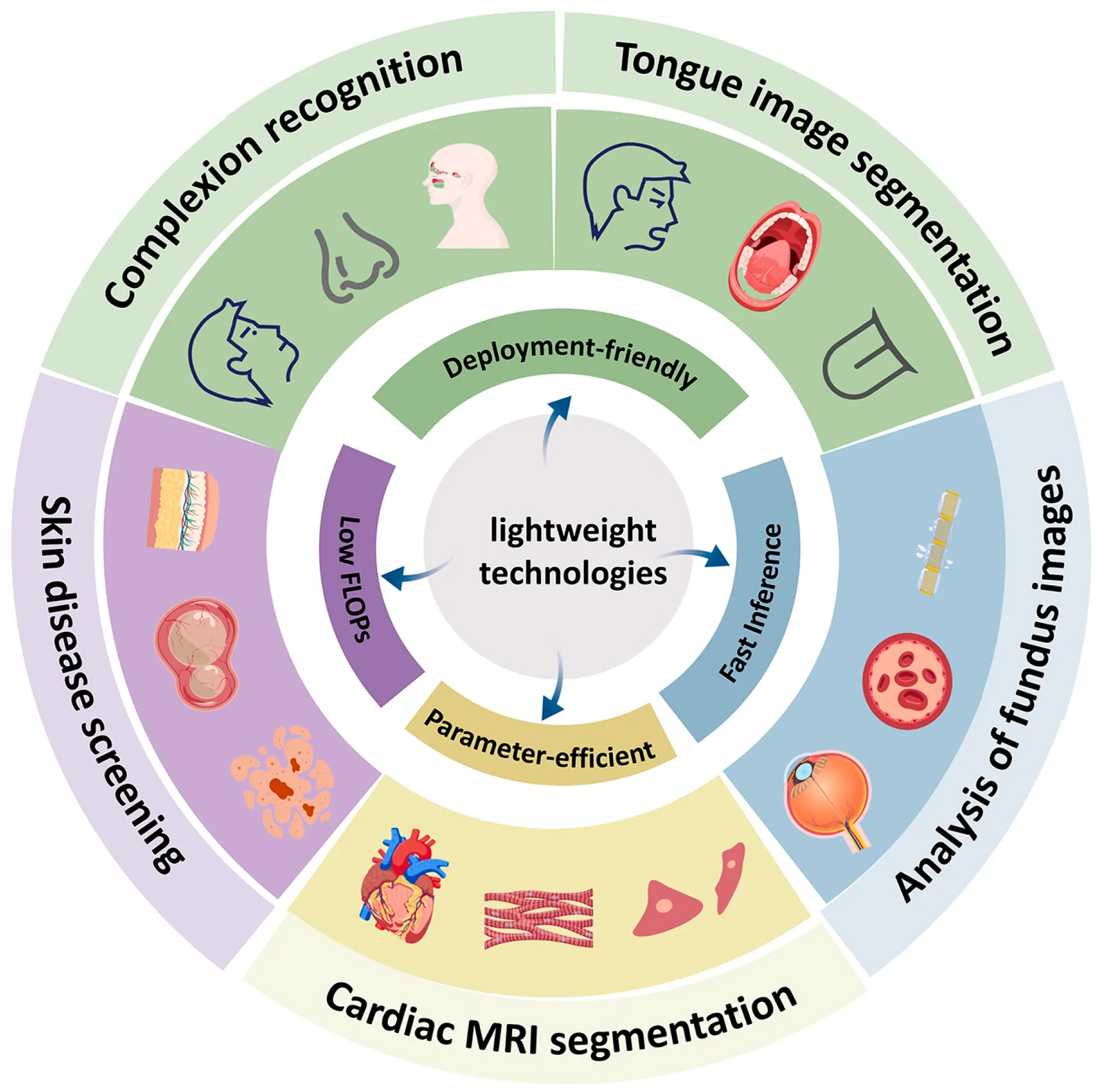
Core characteristics and representative application domains of lightweight neural network technologies.

Lightweight techniques show broad application prospects in the medical field, particularly in resource-constrained edge computing and mobile device scenarios. As shown in Figure 2, in general medical image analysis, lightweight models have been successfully applied to scenarios such as edge computing segmentation of cardiac magnetic resonance imaging (MRI) and smartphone screening of skin lesions [52,53]. In the academic domain, numerous lightweight segmentation models with competitive performance have been developed. For example, within the ultra-lightweight range of only 0.316 M parameters and 0.314 G FLOPs, MK-UNet outperforms existing state-of-the-art methods across six medical imaging benchmarks [54]. Similarly, PUNet integrates the Mamba architecture with depthwise separable convolutions to achieve high-precision real-time segmentation with merely 0.26 M parameters. LM-Net combines the strengths of CNNs and vision transformers (ViTs) to effectively mitigate segmentation boundary blurring, requiring merely 4.66 G FLOPs and 5.4 M parameters [55]. LFT-UNet achieves competitive performance on multiple public datasets with merely 4.469 M parameters, rendering it suitable for embedded deployment [49]. Furthermore, EViT-UNet [52], based on an efficient Transformer; MobileViTv2s [56], based on a linear Transformer; and DyMAS-Net [57], which integrates dynamic multiscale convolutions and dual attention mechanisms, have collectively pushed the boundaries of efficient medical image segmentation.

### 2.3. Lightweight Tongue Diagnosis: From Mobile Systems to Edge Deployment

The widespread use of smartphones has provided a platform for promoting tongue diagnosis. Kanawong et al. developed a mobile application based on computer-aided tongue image analysis technology, using quantitative features to assist doctors in improving the reliability and consistency of diagnosis [58]. Researchers have constructed an automatic tongue diagnosis system utilizing built-in smartphone sensors, in which chromatic deviations under varying illumination conditions are mitigated via a color correction matrix. The correlation between smartphone-captured tongue image features and liver function biomarkers was verified, underscoring the potential of mobile tongue diagnosis for continuous health monitoring [59]. However, an inherent trade-off exists between the large parameter count of deep learning models and the limited computing power of mobile devices: high-precision models are difficult to deploy, and the simple methods that are deployable have insufficient accuracy. This paradox represents a critical bottleneck restricting the clinical translation of AI-driven tongue diagnosis. For instance, Tiryaki et al. employed deep convolutional neural networks for tongue lesion classification, adopting ResNet101 as the backbone and integrating a majority-voting fusion strategy, thereby achieving 95.15% accuracy in binary classification and providing a reliable automated solution for tongue lesion recognition [60]. Zhong et al. addressed the problem of tongue coating color recognition, using DeepLabV3 for tongue body segmentation and then using ResNet50 for classification, attaining 92% accuracy in distinguishing colored from non-colored tongue coatings; notably, the model outperformed the average diagnostic level of three senior traditional Chinese medicine physicians [61].

A growing body of tongue image segmentation models tailored for mobile devices and edge computing devices has emerged. The TongueMobile system achieved automatic tongue segmentation and coating color classification on smartphones with a mean intersection over union of 91% [62]. Qiu et al. proposed a tongue feature extraction method suitable for mobile devices and developed a WeChat Mini Program version of a traditional Chinese medicine tongue diagnosis application [63]. Yang et al. built an intelligent tongue diagnosis system on the Android platform that integrates YOLOv5s6, U-Net, and MobileNetV3 [64]. Song et al. further introduced the concept of edge computing into mobile tongue diagnostic devices, proposing a multi-task network model based on MobileNet [65].

The Mamba architecture, as an efficient alternative to Transformers, has demonstrated tremendous potential in medical image segmentation. The Semi-Mamba-UNet proposed by Ma et al. combines a state-space model with a U-Net to achieve long-range dependency modeling with linear computational complexity, thereby offering a promising architectural alternative for lightweight tongue image segmentation [66]. The Ultralight VM-UNet proposed by Wu et al. significantly reduces the number of parameters while maintaining segmentation accuracy through a parallel visual Mamba scanning mechanism, thereby establishing a viable technical paradigm for embedded tongue diagnosis devices with extremely limited resources [67]. The LightM-UNet proposed by Liao et al. further explores the design paradigm of Mamba-assisted lightweight U-Nets, validating the feasibility of replacing self-attention with state-space models across multiple medical segmentation benchmarks [68].

Recently, researchers have focused more on distilling large models to make them lighter, thereby adapting them to the specific task of tongue image segmentation. For example, TongueSAM_Lite, proposed by Ruan et al., is based on the SAM architecture. TongueSAM_Lite, based on the SAM architecture, reduces model size by 42.7% and shortens inference time through knowledge distillation [45]. MEAT-SAM adapts large models for computationally constrained edge devices such as the Jetson Nano [46]. Tongue-LiteSAM, proposed by Tan et al., employs a dual lightweight ViT-Tiny encoder to achieve competitive zero-shot segmentation capabilities [30]. Recent advancements also include an improved lightweight tongue image segmentation model proposed by Wang et al., which incorporates a self-attention parallel network and progressive upsampling [31], as well as TOM (the first open-source tongue image segmentation tool utilizing multi-teacher distillation and task-specific data augmentation). Using knowledge distillation, this tool reduces the number of parameters by 96.6% while still achieving an mIoU of 95.22% [69]. These works validate the feasibility of high-precision, high-efficiency tongue image segmentation on mobile and edge devices.

## 3. Materials and Methods

### 3.1. Data Collection and Preprocessing

#### 3.1.1. Collection and Screening

The tongue images used in this study were collected from outpatients at Chengdu University of Traditional Chinese Medicine and from individuals undergoing health examinations to encompass diverse tongue morphologies and background complexities, thereby improving model adaptability to real-world scenarios. The dataset encompasses various health conditions and TCM syndromes, including damp-heat, qi deficiency, and yin deficiency, to ensure data diversity and enhance model generalizability. Uniform acquisition protocols and standardized fields of view were established prior to image collection. Standardized tongue-image acquisition devices captured images under fixed illumination conditions, with participants instructed to protrude their tongues naturally, maintain a centered position, and avoid occlusion of the tongue body. The acquisition setup utilized uniform lighting and a fixed background to minimize specular reflections and shadows. To ensure the objectivity and consistency of raw data, the technical parameters of the acquisition devices were standardized in accordance with the latest industry standard for tongue image acquisition in traditional Chinese medicine (YY/T 1488-2025) [70]. Each raw tongue image was assigned a unique identifier that corresponded one-to-one with subsequent annotation files. The raw RGB images served as inputs for deep learning models, whereas versions in HSV and CIELAB (L*a*b*) color spaces were retained for conventional algorithmic processing.

To ensure the quality of training labels and the reliability of experimental conclusions, the collected raw samples were screened using inclusion and exclusion criteria. Inclusion criteria included: the image was required to be clear and well-defined; the main parts of the tongue, such as the tip, edges, and root, were required to be clearly discernible; the tongue must be distinguishable from the background; images must not be overexposed or underexposed; a distinct boundary between the tongue and the background was required; and the resolution met the requirements for subsequent resizing and feature extraction. All images were ultimately resized to 384 × 384 pixels for network input, with a balanced representation of different scenarios, individuals, and tongue appearances to capture a range of health states. Exclusion criteria included severe obstruction of the tongue, extreme blurriness, strong reflections, severe motion blur rendering the tongue margins unrecognizable, and images not depicting the tongue, such as those showing other parts of the mouth or the tongue not protruding. In the construction of medical image datasets, systematic inclusion and exclusion criteria are core steps for ensuring data quality and model reliability.

After sample screening, the data underwent quality cleaning and standardization to meet the requirements for annotation, model training, and experimental reproducibility. Data cleaning comprised three stages. First, file and pairing consistency cleaning: following annotation in LabelMe 5.1 (MIT, Cambridge, MA, USA), JSON files were generated and subsequently converted into binary masks matching the spatial dimensions of the original images via Python 3.9 scripts. The original images and annotation files were required to be in one-to-one correspondence, linked by identical filenames or identifiers; duplicate files were removed, and missing annotations were re-annotated. Second, image-quality cleaning: extremely poor-quality samples were excluded through manual screening based on metrics such as clarity, brightness range, and the proportion of reflective areas; mildly noisy samples were denoised via Gaussian filtering during preprocessing. Third, annotation consistency cleaning: following annotation, boundaries were randomly sampled and reviewed, with particular attention to whether transitional regions at the tongue margin, apex, or base erroneously included lips or teeth. Annotations were restricted to the tongue mucosa, strictly excluding the lips and teeth. Annotations with unclosed contours, substantial misalignments, or severely jagged edges were corrected.

A total of 1000 tongue images were retained after applying inclusion and exclusion criteria. The dataset was randomly partitioned into training (700 images, 70%), validation (150 images, 15%), and test (150 images, 15%) sets at a ratio of 7:1.5:1.5 using a fixed random seed of 42. The three subsets are mutually exclusive, and no image appears in more than one subset. All reported segmentation metrics were computed on the independent test set of 150 images.

#### 3.1.2. Annotation and Preprocessing

All qualified images underwent pixel-level manual annotation using LabelMe. The tongue mucosa boundary was delineated using polygons, with vertices placed sequentially along the mucosal edge; each closed contour was assigned the label “tongue,” thereby generating a JSON file bearing the same filename as the corresponding original image. Subsequently, these JSON files were converted into single-channel binary masks matching the spatial dimensions of the original images using a Python script, with foreground (tongue) pixels encoded as 1 and background pixels as 0.

Data preprocessing was performed differently for traditional segmentation algorithms and deep learning models. Traditional algorithms exhibit sensitivity to color space and illumination conditions, whereas deep learning models impose stricter constraints on input dimensions and data diversity. Gaussian filtering was consistently applied to attenuate high-frequency noise inherent in image acquisition, thereby enhancing the stability of threshold-based segmentation in conventional algorithms and mitigating the risk of noise-induced feature learning in deep learning models. All raw images and corresponding masks were resized to 384 × 384 pixels; bilinear interpolation was employed for raw images, whereas nearest-neighbor interpolation was adopted for masks to preserve categorical boundaries. Deep learning models exclusively utilized RGB images as input.

**Figure 3:**
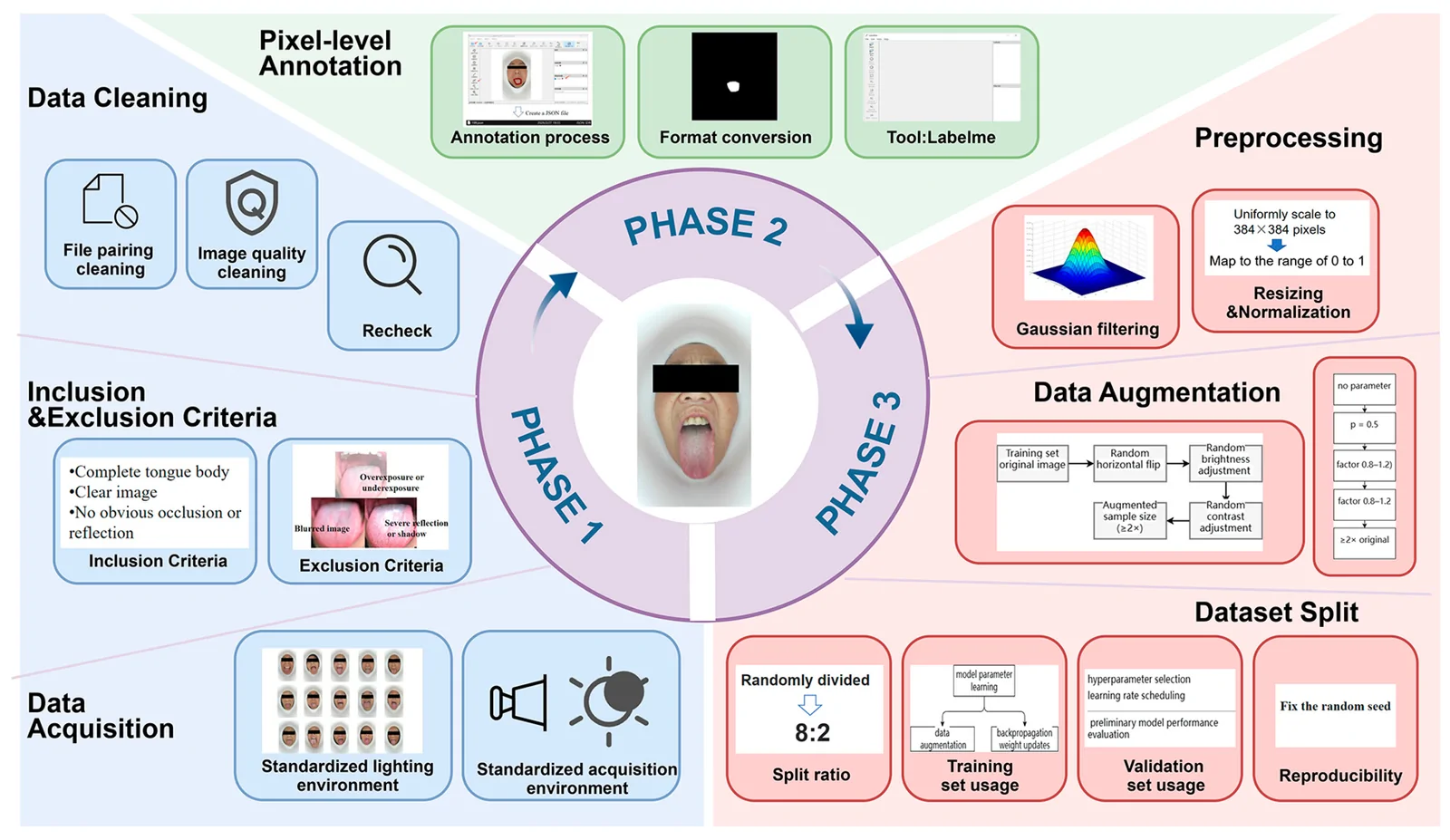
Flowchart of tongue image data acquisition, preprocessing, and dataset partitioning.

By contrast, traditional methods transformed RGB images into HSV and L*a*b* color spaces to facilitate algorithms including Otsu thresholding, GrabCut, and active contour models (snakes). To improve generalization and mitigate overfitting, data augmentation was applied exclusively to the training set, encompassing random horizontal and vertical flipping, brightness adjustment, and contrast adjustment. The augmented training set comprised at least twice as many samples as the original training set. Traditional segmentation algorithms, which operate via pixel-level feature-based automatic segmentation, do not require LabelMe annotation files for algorithmic execution; nevertheless, these annotations were retained as ground truth for quantitative performance evaluation. By contrast, deep learning models required annotation files as supervisory signals for end-to-end training. The overall data preprocessing pipeline, encompassing image collection, screening, quality cleaning, annotation, augmentation, and dataset splitting, is summarized in Figure 3.

### 3.2. Experimental Procedure

To ensure fair comparability between traditional algorithms and deep learning models, this study adopted a unified protocol covering data input, model construction, training, evaluation, and statistical analysis. The experimental pipeline comprised five sequential stages: data preparation, model construction, training optimization, testing and evaluation, and result comparison. The configuration of each stage was designed to satisfy the shared requirements of both methodological paradigms, thereby minimizing confounding variations.

During the data preparation phase, tongue images collected, screened, and cleaned during preliminary processing served as the baseline data. All images underwent standardized preprocessing, including Gaussian filtering for noise reduction, resizing to 384 × 384 pixels, and normalization of pixel values to the range [0, 1]. For deep learning models, the original RGB three-channel images were retained as the primary input to enable the network to learn rich chromatic and textural features. For traditional segmentation algorithms, which are sensitive to variations in color space and illumination, images were additionally transformed into the HSV and L*a*b* color spaces to facilitate color-based region extraction and boundary optimization. After preprocessing, all samples were randomly split into training, validation, and test sets at a ratio of 7:1.5:1.5 using a fixed random seed of 42 to ensure experimental reproducibility. Specifically, 700 images were allocated to the training set, 150 images to the validation set, and 150 images to the independent test set. The training set was employed for model parameter learning and data augmentation; the validation set was used for hyperparameter selection and learning rate scheduling; and the independent test set was used for final performance evaluation. For traditional algorithms, which do not involve learnable parameters, the independent test set was used for inference-based performance evaluation.

A dual-path parallel design was adopted during the model-building phase. One path consisted of traditional image segmentation methods, with GrabCut and active contour models (snakes) selected as baseline algorithms. Both algorithms were tailored to the characteristics of tongue images, including complex backgrounds, blurred edges, and chromatic similarity.

For GrabCut, the initial foreground region was automatically generated in the L*a*b* color space, thereby transforming the interactive algorithm into a fully automated segmentation process. The number of iterations was set to 8, and both the background and foreground models used default initializations. For Snake, image preprocessing was performed using a Gaussian filter with a standard deviation of σ = 3 to attenuate noise. The contour of the largest connected component in the automatically generated initial mask was adopted as the initialization. To accommodate the soft edges of the tongue, the rigidity coefficient was reduced to increase flexibility: the tension coefficient α was set to 0.015, the rigidity coefficient β to 10, and the gradient weight to 1. The external energy was derived primarily from the image gradient, and the contour evolution underwent 20 iterations.

The other path comprised deep learning-based segmentation models. Eight representative lightweight semantic segmentation networks were selected, including UNet++, SegNeXt-T, Swin-Tiny, HRNet, OCRNet, BiSeNet, PP-LiteSeg, and SegFormer-B1. These models incorporated mainstream architectural paradigms, including multiscale feature fusion, high-resolution representation preservation, dual-branch real-time inference, and lightweight Transformer blocks. All models were adapted for lightweight implementation based on their original architectures. Key strategies included channel reduction, depth reduction, replacement of standard convolutions with depth-separable convolutions, simplification of feature fusion modules, and replacement of backbone networks, rendering the models suitable for tongue image segmentation under resource-constrained conditions. Table 1 summarizes the key modifications of each model relative to its original architecture.

In the training and optimization stage, deep learning models were trained in a consistent computational environment. During training, the tongue binary masks derived from manual annotations served as supervision, and model parameters were optimized through backpropagation to align predictions with ground-truth annotations. The training set was used for parameter learning and data augmentation; the validation set was employed to monitor generalization performance, guide hyperparameter selection, and inform learning rate scheduling. To mitigate overfitting arising from limited data, the training set was augmented through random horizontal and vertical flipping as well as brightness and contrast adjustments. Specifically, the horizontal flip probability was set to 0.5, and both brightness and contrast factors were sampled uniformly from [0.8, 1.2]. Data augmentation expanded the training set to at least twice its original size.

Regarding optimization, most models employed AdamW with an initial learning rate of 1 × 10^−3^ and a weight decay of 1 × 10^−4^; UNet++ and Swin-Tiny utilized the Adam optimizer at the same learning rate. Except for PP-LiteSeg, which employed a combined loss of Dice loss and binary cross-entropy with logits (BCEWithLogitsLoss), all other models employed BCEWithLogitsLoss. The number of training epochs was determined based on convergence speed: BiSeNet, HRNet, OCRNet, PP-LiteSeg, SegFormer-B1, and UNet++ were trained for 50 epochs, whereas SegNeXt-T and Swin-Tiny were trained for 100 epochs. Batch size was determined according to available GPU memory. UNet++ adopted a batch size of 4 owing to its complex architecture and multilayer feature fusion, which incurred high GPU memory consumption; all remaining models used a batch size of 8. All models were trained with automatic mixed precision via torch.cuda.amp (autocast and GradScaler) to reduce GPU memory footprint and maintain numerical stability. All models were trained with a fixed random seed of 42 for the main experiments. For the four top-performing models (UNet++, BiSeNet, PP-LiteSeg, and HRNet), additional training runs with seeds 123 and 2024 were performed to estimate 95% confidence intervals.

Model checkpointing was performed with the validation Dice coefficient as the monitoring metric; model parameters were saved whenever the Dice score improved. UNet++ utilized TensorBoard to record loss trajectories as well as training and validation Dice scores, facilitating monitoring of training dynamics and hyperparameter tuning. PP-LiteSeg additionally employed the ReduceLROnPlateau scheduler to decay the learning rate to 50% of its current value when the validation Dice showed no improvement over 5 epochs. All other models maintained a fixed learning rate.

For traditional algorithms, as they do not involve an iterative training process, segmentation was achieved through parameter tuning and pipeline optimization alone. These algorithms served as fixed benchmarks for subsequent evaluation, without any training or parameter updates.

In the evaluation stage, all models were assessed on the same hardware platform under identical conditions to ensure fair comparison. Model parameters were fixed, and the independent test set images were fed into each method to obtain the corresponding tongue segmentation results. The hardware platform was an NVIDIA RTX 3050 Ti (NVIDIA, Santa Clara, CA, USA) mobile GPU (NVIDIA Corporation, Santa Clara, CA, USA), the processor was an AMD Ryzen 6800H (Advanced Micro Devices, Inc., Santa Clara, CA, USA), and the software environment comprised PyTorch 1.12(Meta AI, New York, NY, USA) and PaddlePaddle 2.4 (Baidu, Beijing, China). The input image size was uniformly set to 384 × 384 pixels, and each model was inferred 10 times consecutively to compute the average, thereby minimizing measurement variance. The models were subsequently evaluated comprehensively in terms of segmentation accuracy and operational efficiency. Accuracy metrics comprised the mean Intersection over Union (mIoU), which quantifies the overlap between predicted and ground-truth regions; mean pixel accuracy (mPA), which reflects overall pixel classification accuracy; and the Dice similarity coefficient, which evaluates the similarity between predicted and ground-truth regions and is more sensitive to edge quality. Efficiency metrics included single-image inference time and frames per second (FPS), reflecting inference latency and throughput, respectively. The advantages and disadvantages of traditional algorithms and deep learning models for tongue image segmentation were compared under a unified evaluation framework.

The following sections detail the implementation of the traditional segmentation algorithms and deep learning models, respectively.

#### 3.2.1. Methods Based on Traditional Image Segmentation

Early research on tongue image segmentation primarily focused on traditional image processing methods. Pang et al. proposed a segmentation method combining a double elliptical deformation template with an active contour model. By employing an energy function, the method captured global shape features of the tongue and achieved satisfactory segmentation performance under controlled imaging conditions [71]. Shi et al. combined a geometric Snake model with a parametric gradient vector flow (GVF) Snake model, incorporating color space information to guide contour evolution and thereby enabling automatic tongue image segmentation. In the same year, the same group further proposed a dual geometric vector flow method for tongue boundary detection and regional segmentation, demonstrating improved performance [72]. Nevertheless, these traditional methods exhibited limited robustness under uncontrolled clinical conditions. They are sensitive to changes in lighting, making it difficult to effectively distinguish the tongue from similar color areas such as the lips. Additionally, Yan et al. applied GrabCut guided by visual saliency to sublingual mass segmentation, in which GrabCut was first initialized using saliency information to extract the tongue region, followed by Otsu multi-thresholding for target refinement [73]; Zhao et al. used the GrabCut method to segment the tongue in the study of cracked tongue recognition, extracting three types of features (texture, color, and shape) and inputting them into a machine learning model, achieving a recognition effect almost equivalent to that of deep learning models [74]. Collectively, these studies demonstrated that, although traditional algorithms achieved significant progress, their performance remained limited to semi-automatic segmentation. For challenging cases, manual intervention was often required to achieve satisfactory segmentation; moreover, the computational efficiency of these methods was generally insufficient to meet the real-time requirements of portable devices.

Traditional image segmentation methods rely on handcrafted low-level features, such as color distributions and edge cues. Although these methods do not require training data and incur relatively low computational costs, their robustness remains limited under clinical conditions characterized by blurred tongue boundaries, uneven illumination, and complex backgrounds. To quantify the performance improvement achieved by deep learning methods, the present study selected two representative traditional segmentation algorithms as benchmarks: the GrabCut graph-cut algorithm and the active contour model (Snake). These algorithms represent typical region- and boundary-based techniques, respectively, and served as rigorous baselines for subsequent comparison with deep learning models.

The core idea of the GrabCut algorithm is to formulate image segmentation as a binary labeling optimization problem based on graph theory. The algorithm establishes Gaussian mixture models (GMMs) for foreground and background color distributions, each comprising multiple Gaussian components to account for color distribution diversity. Segmentation is subsequently optimized via iterative energy minimization. The energy function comprises two terms: a data term that encodes the likelihood of a pixel’s color belonging to the foreground or background model, and a smoothing term that enforces spatial coherence among neighboring pixel labels and promotes label consistency among chromatically similar pixels. To achieve fully automatic segmentation without manual interaction, the present study employed the L*a*b* color space to generate the initial foreground region. Specifically, the initial mask was obtained by applying channel-wise thresholds in L*a*b* space: regions within the mask were marked as probable foreground, and the remaining regions as background. Iterative optimization was then performed; each iteration consisted of three steps—component assignment, parameter estimation, and graph-cut-based segmentation—and the procedure was repeated for a total of eight iterations. Finally, the max-flow min-cut algorithm was employed to generate the binarized tongue region. Both foreground and background models were initialized using default parameters.

The Snake model, also known as the active contour model, drives an initial contour to evolve toward the target boundary by minimizing a contour energy function. The model represents the target contour as a parameterized curve; the total energy comprises internal and external terms. The internal energy governs the physical properties of the contour: the tension term penalizes contour stretching, whereas the rigidity term penalizes bending. The flexibility of the contour is adjusted by varying the tension coefficient α and the rigidity coefficient β. To accommodate the soft edges of the tongue, the rigidity coefficient was reduced to increase contour flexibility.

The external energy is derived from image data and is typically formulated as a negative function of the image gradient magnitude, such that the external energy reaches minima at strong edges and attracts the contour toward the target boundary. To eliminate the need for manual initialization in the conventional Snake model, the contour of the largest connected component in the automatically generated initial mask was adopted as the initial contour. Contour evolution was solved iteratively via gradient descent. At each step, internal and external forces were computed at the current contour points, and the points were updated to reduce the total energy. A total of 20 iterations were performed. Image preprocessing employed Gaussian filtering with a standard deviation of σ = 3 to attenuate noise. Regarding parameter configuration, the tension coefficient α was set to 0.015, the rigidity coefficient β to 10, and the gradient weight to 1, with the image gradient serving as the dominant external energy term.

Both traditional algorithms were evaluated on the tongue image dataset constructed in the present study, and the segmentation results served as benchmarks for subsequent comparison with deep learning models.

#### 3.2.2. Methods Based on Deep Learning for Image Segmentation

This study selected eight representative lightweight semantic segmentation models, covering mainstream architectural paradigms including classic encoder–decoder, high-resolution representation preservation, dual-branch real-time inference, and lightweight Transformer architectures. Specifically, these models included UNet++ [75], SegNeXt-T [25], Swin Tiny [76], HRNet [77], OCRNet [78], BiSeNet [79], PP-LiteSeg [24], and SegFormer-B1 [26]. These models were selected because they represented paradigmatic examples of their respective architectural classes, and their publicly available lightweight implementations were amenable to deployment on resource-constrained devices by virtue of their favorable parameter-efficiency trade-offs. Fair comparison of these models on a unified platform enabled a comprehensive assessment of the state of the art in lightweight segmentation and its adaptability to tongue image segmentation.

To ensure effectiveness and efficiency for tongue segmentation, all networks underwent lightweight adaptation and task-specific modifications relative to their original architectures. Key strategies included channel reduction to decrease parameter counts, depth reduction to decrease the number of repeated blocks across stages, replacement of standard convolutions with depthwise separable or grouped convolutions to reduce multiply–accumulate operations, simplification of feature fusion modules (e.g., replacing channel concatenation with element-wise addition followed by convolution), and replacement of computationally intensive backbones with MobileNetV2 or custom lightweight encoders. Table 1 summarizes the key modifications of each model relative to its original architecture.

The original UNet++ architecture employs nested dense skip connections and deep supervision to mitigate the semantic gap between the encoder and decoder. In this work, the nested U-shaped topology was retained with two lightweight modifications: the deep supervision branches were removed, leaving only a single output head, and the channel dimensions were reduced to 32, 64, 128, 256, and 512, yielding a more compact configuration than the standard implementation. Each building block followed a VGG-style design, comprising two successive 3 × 3 convolutional layers, each followed by batch normalization and a Rectified Linear Unit (ReLU) activation. These modifications substantially decreased the parameter count and computational overhead while preserving multi-scale feature fusion capabilities. For single-object segmentation tasks—such as tongue image analysis—the incremental benefit conferred by deep supervision is marginal.

The principal innovation of SegNeXt-T resides in its multi-scale convolutional attention (MSCA) module. The original implementation employs parallel large-kernel depthwise convolutions with varying dilation rates to capture multi-scale contextual information. Here, it was simplified into a serial architecture: a 5 × 5 depthwise convolution extracted local spatial features, followed by sequential 1 × 7 and 7 × 1 strip convolutions to model long-range dependencies, and a final 1 × 1 pointwise convolution for channel mixing. The decoder eschewed complex stage-wise upsampling and fusion; instead, backbone features from all four stages were unified to a common spatial resolution, concatenated, and subsequently fed into a lightweight convolutional head. This reconfiguration transformed the multi-scale attention mechanism from a parallel to a serial topology, thereby reducing computational complexity. Moreover, the extensive receptive field of the strip convolutions effectively spans the long-axis orientation of the tongue, which is critical for preserving the spatial coherence of the tongue coating distribution.

The original Swin-Tiny model employs a hierarchical transformer architecture with shifted-window self-attention. Although its computational complexity scales linearly with image resolution, it remains suboptimal for resource-constrained, mobile-edge deployment. In this work, a purely convolutional lightweight variant was implemented: patch embedding was performed via a convolutional layer with a stride of 4; each subsequent stage combined grouped convolutions and 1 × 1 pointwise convolutions to approximate the channel-wise grouping behavior of multi-head attention; and strided convolutions with a stride of 2 were adopted for inter-stage downsampling. This variant discarded window attention and relative position bias entirely, substituting the underlying transformer blocks with efficient convolutional operators. While its global context-modeling capacity is marginally inferior to that of the standard Transformer, inference throughput improved by an order of magnitude when processing uniformly illuminated standard tongue images. Moreover, the hierarchical feature extraction capability was preserved.

The original HRNet architecture preserves high-resolution spatial details through parallel multi-resolution branches with repeated cross-resolution information exchange. The LightHRNet18 adopted herein substantially streamlined this design: the parallel multi-branch topology was replaced by a single-branch sequential structure, yet a quarter-resolution feature map derived from the original image was maintained throughout the network following the initial trunk. Channel dimensions were progressively expanded from 16 to 32, 64, and 128 across successive stages. Each building block followed the BasicBlock design, comprising two successive 3 × 3 convolutions with residual connections. Although this simplified configuration sacrifices the full representational capacity of multi-branch fusion, it retains HRNet’s core emphasis on precise spatial localization. Consequently, the overhead associated with multi-branch maintenance and cross-resolution feature interaction was markedly reduced, rendering the architecture better suited for tasks—such as tongue image segmentation—that demand fine edge-detail preservation without reliance on large-scale, multi-object contextual modeling.

The original OCRNet employs high-capacity backbones—such as HRNet—for feature extraction. Pixel representations are enhanced via an object context module that performs multi-region soft segmentation and pixel-wise computation of relational matrices across object regions. In this work, a lightweight adaptation was proposed: the backbone was replaced by MobileNetV2, whereby the parameter count was substantially reduced through inverted residual blocks and depthwise separable convolutions; the object context module was streamlined into a minimalist OCRBlock. Specifically, object representation vectors were first generated via global average pooling and 1 × 1 convolutions, then modulated through element-wise multiplication with per-pixel feature vectors, and subsequently activated by a Sigmoid function. For tongue image segmentation, substituting multi-region soft segmentation with a global semantic representation furnished adequate contextual constraints, effectively filling internal voids caused by coating peeling or mapping artifacts while incurring minimal computational overhead.

The original BiSeNet adopts a two-branch architecture in which the spatial pathway preserves high-resolution details and the context pathway rapidly downsamples to acquire a large receptive field; bilateral features are subsequently fused through a complex guided aggregation module. This work retained the dual-branch paradigm but simplified both pathways and the fusion mechanism: the detail branch comprised three convolutional stages, retaining features at 1/4 resolution; the semantic branch consisted of a Stem module followed by three rapid downsampling stages, culminating in a 1/32-resolution feature map; feature fusion was performed via the simplest element-wise addition without resorting to additional attention or gating mechanisms. This architecture was explicitly optimized for real-time inference. The detail branch accurately delineated the tongue contour, whereas the semantic branch coarsely localized the tongue region, collectively enabling high-throughput inference on embedded devices.

The original PP-LiteSeg employs a Short-Term Dense Concatenate (STDC) backbone, a Unified Attention Fusion Module (UAFM), and a lightweight feature pyramid. Rather than directly adopting the STDC network, this work designed a custom lightweight encoder with an analogous topology that extensively utilized strided and depthwise separable convolutions. Channel dimensions were configured as 32, 64, 128, and 256, with a width multiplier halving the channel count to further curtail computational overhead. The decoder retained the UAFM, which comprised two components: channel attention and spatial attention. High-level features were first upsampled via bilinear interpolation and subsequently modulated by channel attention; low-level features were recalibrated by spatial attention, and the two branches were aggregated via element-wise addition. For tongue image segmentation, channel attention accentuated chromatic semantics critical to tongue identification, whereas spatial attention reinforced edge and tooth-mark textures in shallow layers, thereby achieving high-precision boundary delineation with a minimal parameter budget.

The original SegFormer-B1 architecture comprises a hierarchical MixVisionTransformer encoder and a lightweight multilayer perceptron (MLP) decoder; it omits positional encoding, thereby exhibiting inherent robustness to variations in input resolution. This work preserved the overall framework but emphasized training-efficiency adaptations: the encoder employed overlapped patch embeddings across four stages, with spatial reduction ratios in self-attention progressively set to 8, 4, 2, and 1. Keys and values were spatially downsampled to reduce computational complexity. The decoder was highly streamlined: multi-stage outputs were projected to 256 channels via 1 × 1 convolutions, upsampled to a uniform spatial resolution, and concatenated. The final segmentation mask was produced directly by an MLP layer. For tongue image segmentation, the model leveraged global attention to discriminate the tongue from chromatically similar labial and oral mucosal regions, and maintained stable segmentation performance despite variations in tongue protrusion posture.

All models received an input resolution of 384 × 384 pixels, with standardized preprocessing, training strategies, and evaluation metrics. Fair comparisons were ensured by conducting all experiments on an identical hardware platform and dataset.

#### 3.2.3. Training Strategies and Hyperparameter Settings

All deep learning models were trained under a unified experimental setup to ensure fair comparison and reproducibility. Input images were uniformly resized to 384 × 384 pixels, and pixel values were normalized to the range [0, 1]. For the optimizer, most models adopted AdamW with an initial learning rate of 1 × 10^−3^ and a weight decay of 1 × 10^−4^; UNet++ and Swin-Tiny used the Adam optimizer with the same learning rate. For SegFormer-B1, a lower initial learning rate of 1 × 10^−4^ was used for stable training. Regarding the loss function, except for PP-LiteSeg, which employed a combination of Dice loss and BCEWithLogitsLoss, all other models used BCEWithLogitsLoss alone. The Dice loss is computed as 1 minus twice the intersection of the predicted and ground-truth pixels, divided by the sum of their pixel counts. When combined with the cross-entropy loss, it simultaneously optimizes both region overlap and pixel-wise classification accuracy, which is particularly beneficial for delineating the tongue edges.

The number of training epochs varied slightly according to the convergence speed of different models. BiSeNet, HRNet, OCRNet, PP-LiteSeg, SegFormer-B1, and UNet++ were trained for 50 epochs, whereas SegNeXt-T and Swin-Tiny were trained for 100 epochs. The batch size was set based on memory constraints; UNet++ used 4, and the other models used 8. The hardware platform was an NVIDIA RTX 3050 Ti mobile GPU.

During training, all models used automatic mixed precision to reduce GPU memory usage and maintain numerical stability. Model evaluation and checkpointing used the Dice coefficient on the validation set as the primary monitoring metric and saved the current model parameters whenever the Dice score improved. UNet++ used TensorBoard to record losses and Dice changes. PP-LiteSeg additionally used the ReduceLROnPlateau learning rate scheduler to decay the learning rate by a factor of 0.5 when the validation set Dice did not improve within 5 epochs. The remaining models used a fixed learning rate. The detailed training parameter settings of each model are summarized in Table 2.

### 3.3. Evaluation Indicators

Model performance was evaluated across two dimensions: segmentation accuracy and computational efficiency. Segmentation accuracy was quantified using mean Intersection over Union (mIoU) and mean pixel accuracy (mPA). Computational efficiency was assessed in terms of inference latency and throughput (frames per second, FPS). Efficiency metrics were reported as the average over the independent test set of 150 images.

Segmentation quality was further analyzed using a confusion matrix. In this binary segmentation task, the tongue region constituted the foreground, with all other regions designated as background. Pixel-wise comparison between the predicted mask and the ground-truth annotation yielded four categories of outcomes: true positives (TP), correctly classified tongue pixels; false positives (FP), background pixels misclassified as tongue; false negatives (FN), tongue pixels misclassified as background; and true negatives (TN), background pixels correctly classified as background. From these quantities, the following metrics were derived.

As shown in Equation (1), mIoU is defined as the average intersection-over-union ratio between the predicted and ground-truth regions:

(1) $$\begin{array}{c} mIoU=\frac{1}{N}\sum_{i=1}^{N}\frac{{TP}_{i}}{{TP}_{i}+{FP}_{i}+{FN}_{i}} \end{array}$$

where N denotes the total number of categories and i is the category index, and TP_i_, FP_i_, and FN_i_ represent the true positives, false positives, and false negatives for category i, respectively. In this task, N = 2, corresponding to the tongue and background.

The mPA, defined in Equation (2), measures the average proportion of correctly classified pixels across all categories, reflecting overall segmentation accuracy:

(2) $$mPA=\frac{1}{N}\sum_{i=1}^{N}\frac{{TP}_{i}}{{TP}_{i}+{FP}_{i}}$$

where N and i are as defined in Equation (1), and TP_i_ and FP_i_ denote the true positives and false positives for category i, respectively.

FPS was computed from the inference time, as shown in Equation (3):

(3) $$FPS=\frac{1000}{Inference\;Time\;(\mathrm{ms})}$$

where the constant 1000 represents the conversion factor from milliseconds to seconds, and Inference Time denotes the per-image inference latency measured in milliseconds (ms).

Based on the above datasets, models, and evaluation metrics, this study conducted a systematic comparative analysis. First, the quantitative performance of two traditional segmentation algorithms and eight lightweight deep learning models on the tongue image segmentation task was compared. Subsequently, the differences among the models were further examined at three levels: qualitative segmentation results, comprehensive performance comparison, and detailed metric analysis.

## 4. Results

### 4.1. Quantitative Result Comparison

The quantitative results of the two conventional segmentation algorithms and the eight deep learning models are summarized in Table 3 and Table 4, respectively. As presented in Table 3, the mIoU values of GrabCut and Snake were comparable (0.8684 and 0.8673, respectively), as were their mPA values (0.8803 and 0.8831, respectively), indicating that both methods enabled the fundamental segmentation of the tongue region. Nevertheless, the two methods exhibited substantial differences in computational efficiency. Specifically, GrabCut required an inference time exceeding 10 s, whereas Snake required approximately 2 s; consequently, neither method achieved an FPS exceeding 0.5.

As shown in Table 4, four deep learning models attained mIoU values above 0.94, with OCRNet showing the lowest performance (mIoU = 0.9033). The full training, validation, and test performance is provided in Supplementary Table S1, showing no severe overfitting. Among the evaluated models, UNet++ achieved the highest mIoU (0.9543), followed by BiSeNet (0.9484), HRNet (0.9467), and PP-LiteSeg (0.9462). BiSeNet achieved the fastest inference speed (310.03 FPS) together with the highest mPA (0.9766), making it the most efficient model for real-time deployment. HRNet achieved the second-fastest inference speed (272.64 FPS) with a competitive mIoU (0.9467), positioning it as a strong candidate for high-resolution, real-time tongue segmentation. SegNeXt-T and SegFormer-B1 achieved moderate mIoU values (0.9300 and 0.9299, respectively), while Swin-Tiny and OCRNet showed the lowest performance among the evaluated models. In contrast, despite its relatively high accuracy, UNet++ incurred a considerably longer inference time (12.40 ms, 80.65 FPS), rendering it more suitable for offline or server-side analysis rather than real-time applications. Overall, relative to the traditional algorithms, the deep learning-based methods improved the mIoU by approximately 9% and increased the inference speed by more than two orders of magnitude. The training convergence curves are provided in Supplementary Figure S1, and the training and validation Dice curves are shown in Supplementary Figure S2. The train–validation gap remains small for most models, indicating no severe overfitting. To assess the statistical significance of the observed differences, we trained the four top-performing models (UNet++, BiSeNet, PP-LiteSeg, and HRNet) with 3 random seeds (42, 123, 2024) and report the mean ± 95% confidence interval in Table 5. UNet++ shows the smallest CI width (±0.0011 for Dice and ±0.0022 for mIoU), indicating highly stable training. To further verify whether the performance differences between models are statistically significant, we performed a paired bootstrap analysis (10,000 resamples) on the per-image Dice scores of seed 42 for each model (Table 6). UNet++ significantly outperforms BiSeNet (ΔDice = +0.0031, 95% CI [+0.0023, +0.0038]), PP-LiteSeg (ΔDice = +0.0043, 95% CI [+0.0031, +0.0056]), and HRNet (ΔDice = +0.0040, 95% CI [+0.0030, +0.0052]), as all confidence intervals lie entirely above zero. These results confirm that the performance advantage of UNet++ is statistically significant under the unified test protocol.

### 4.2. Analysis of Qualitative Results

**Figure 4:**
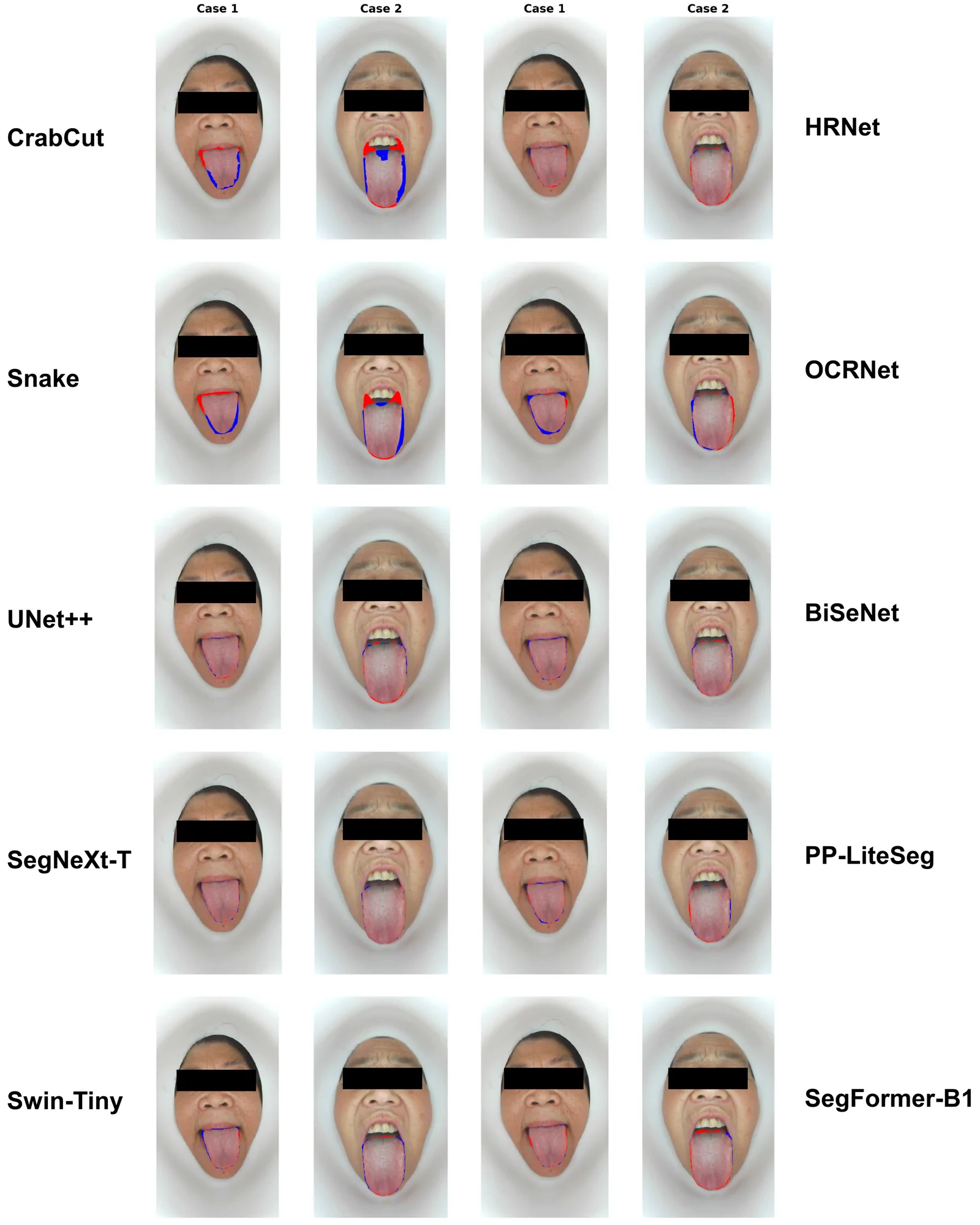
Qualitative comparison of segmentation results for 10 algorithms on two representative tongue images (Case 1 and Case 2). Red, false negatives (missed tongue region); blue, false positives (misclassified background).

The qualitative performance of each algorithm was further evaluated in terms of visual fidelity and error patterns. Figure 4 presents a comparative visualization of the segmentation results obtained by the ten algorithms on two representative tongue images (Case 1 and Case 2). Red regions indicate false negatives (missed tongue regions), while blue regions indicate false positives (background misclassified as tongue). Zoom-in boxes highlight the tongue tip, lateral margin, and tongue base, where most segmentation errors occur.

Case 1: Blurred tongue–lip boundary. The tongue tip contacts the lower lip, and both regions show similar color under non-uniform illumination. GrabCut and Snake produce severe errors: GrabCut shows false negatives at the tongue base and false positives at the lateral margin; Snake over-segments beyond the true boundary. Among deep learning models, UNet++, BiSeNet, HRNet, and PP-LiteSeg achieve accurate segmentation with minimal boundary deviation. SegNeXt-T and SegFormer-B1 show moderate performance with slight lateral deviations. Swin-Tiny produces dispersed false negatives within the tongue body. OCRNet shows the largest deviation, consistent with its lowest mIoU (0.9033).

Case 2: Thick coating with partial occlusion. The tongue surface is covered by a thick coating, and the tongue base is partially occluded by oral shadow. Traditional methods show large missed regions at the tongue base and misclassified regions along the lateral margin. Among deep learning models, BiSeNet, HRNet, and PP-LiteSeg maintain accurate segmentation at the tongue base and coated region. UNet++ achieves the highest overall mIoU (0.9543) but shows a localized false-negative region in the thick coating area. SegNeXt-T and SegFormer-B1 show slight boundary deviations. Swin-Tiny and OCRNet produce more pronounced false negatives in the coating region.

Overall, deep learning models consistently outperform traditional methods. BiSeNet, HRNet, and PP-LiteSeg are the most robust across both cases. UNet++ achieves the highest quantitative accuracy but shows a localized failure under thick coatings, revealing a trade-off between global accuracy and robustness to low-contrast boundaries.

**Figure 5:**
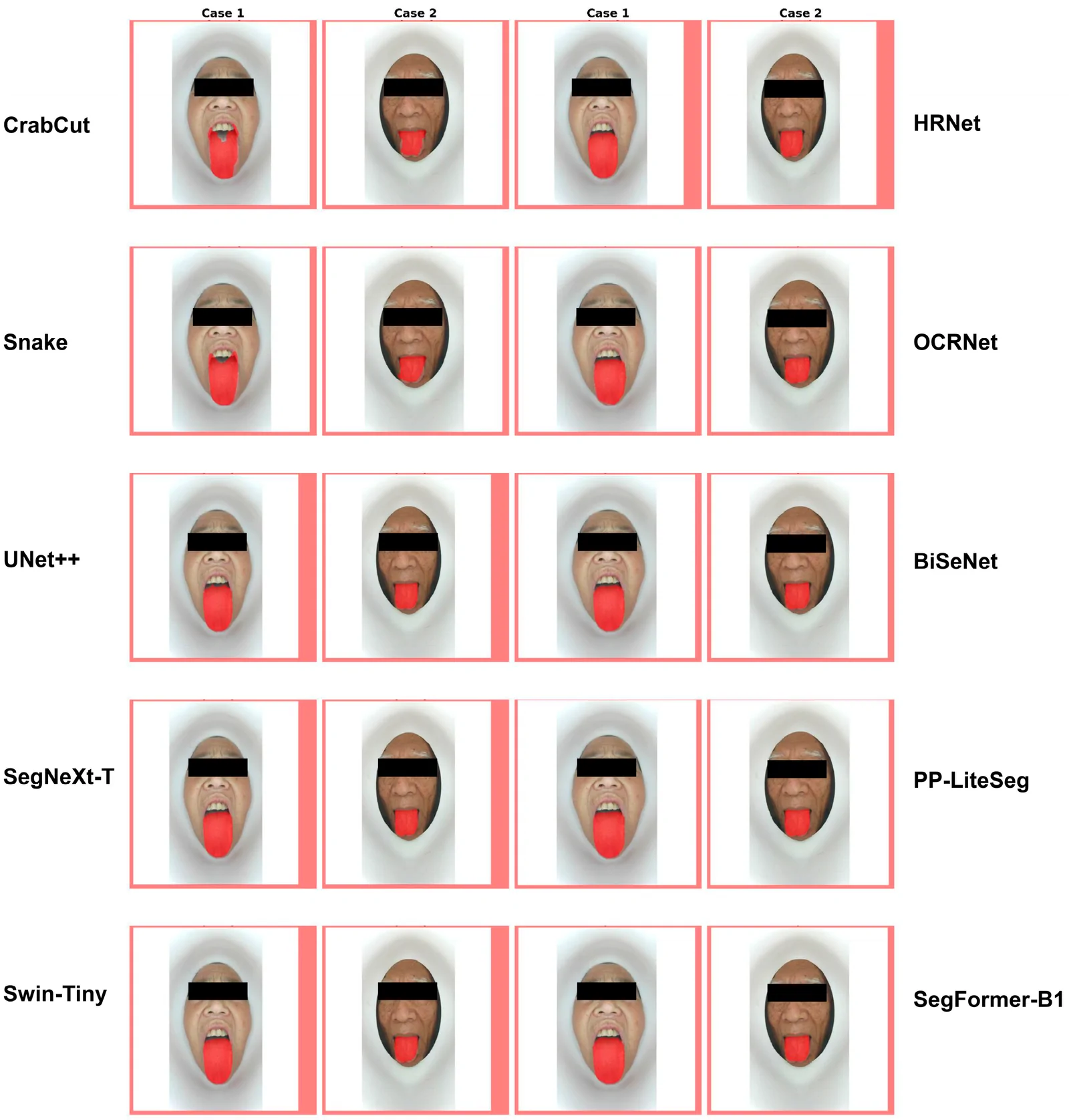
Visual segmentation outputs of comparative algorithms on tongue images. Red overlays indicate the foreground (tongue region) extracted via binary masking.

Figure 5 illustrates the visual segmentation outcomes of the evaluated algorithms on two additional challenging cases. Red overlays indicate the segmented tongue region. In Case 1, the tongue tip is in close contact with the lower lip under non-uniform illumination, and the chromatic characteristics of the two regions are highly similar. Conventional methods (GrabCut and Snake) exhibited marked over-segmentation at the lateral margin and under-segmentation at the tongue base. Among the deep learning models, UNet++, BiSeNet, HRNet, and PP-LiteSeg maintained complete tongue masks with minimal boundary deviation, whereas SegNeXt-T and SegFormer-B1 showed slight boundary deviations near the tongue tip, and Swin-Tiny and OCRNet produced dispersed false negatives within the tongue body.

In Case 2, the tongue surface is covered by a thick coating, and the tongue base is partially occluded by oral shadow, making the boundary between the tongue and the oral cavity difficult to distinguish. Conventional methods produced large false-negative regions at the tongue base and false-positive regions along the lateral margin. Among the deep learning models, BiSeNet, HRNet, and PP-LiteSeg preserved accurate segmentation in the coated region and at the tongue base, whereas SegNeXt-T and SegFormer-B1 showed minor boundary deviations, and Swin-Tiny, OCRNet, and UNet++ exhibited localized false negatives in the coating region. Overall, deep learning models consistently outperformed conventional methods across both cases, with BiSeNet, HRNet, and PP-LiteSeg demonstrating the most robust performance, while UNet++ achieved the highest global accuracy but showed a localized failure under thick coatings.

### 4.3. Comprehensive Performance Evaluation

**Figure 6:**
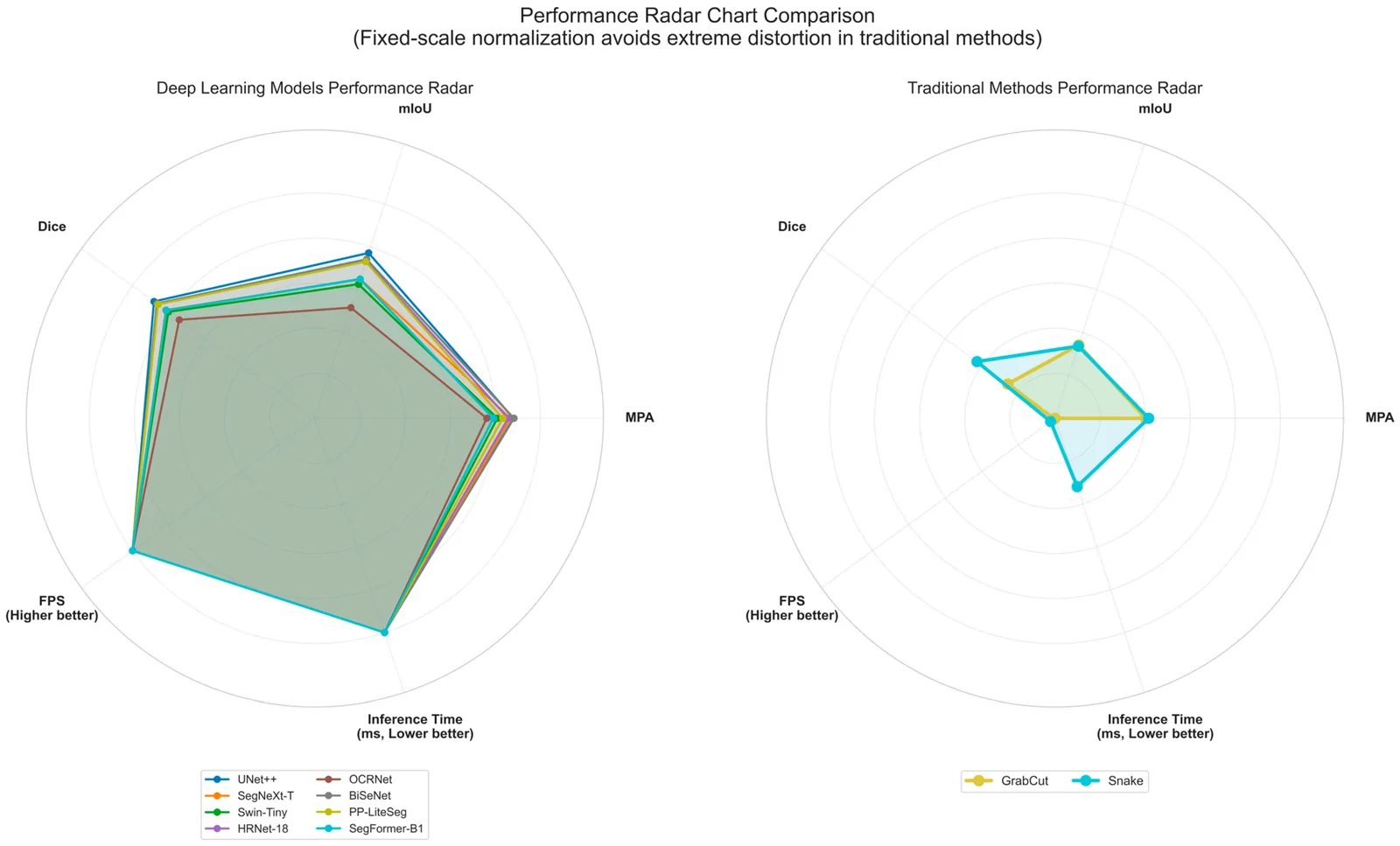
Radar-chart comparison of model performance across mIoU, Dice, mPA, FPS, and inference time. A larger enclosed area indicates superior overall performance.

**Figure 7:**
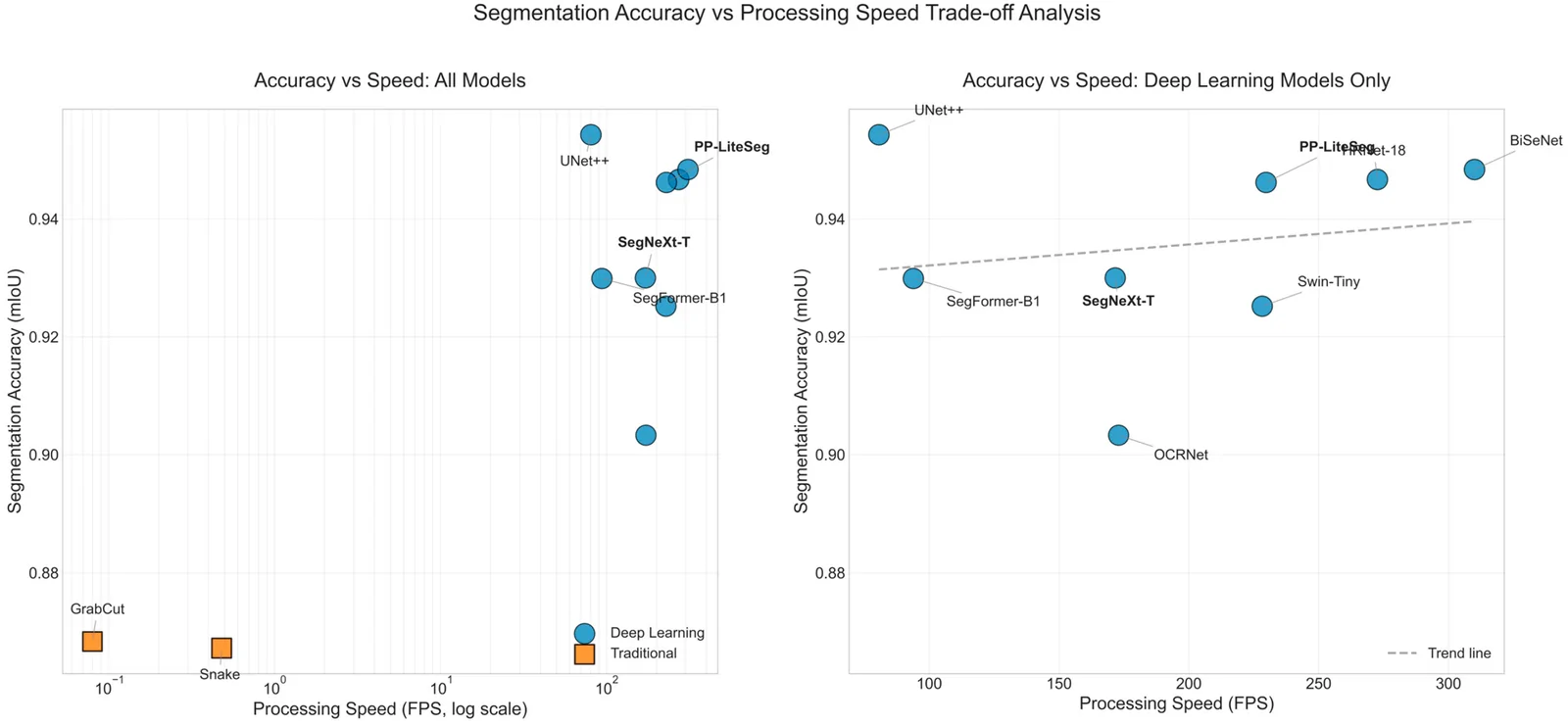
Scatter plot of segmentation accuracy (mIoU) versus inference time (logarithmic horizontal axis). Each data point corresponds to a specific model, with model identifiers annotated adjacent to the respective point.

To facilitate a holistic comparison of model performance, radar charts, scatter plots, and heatmaps were employed to visualize the multi-dimensional metrics. Figure 6 presents a radar chart comparing the ten algorithms across five core indicators: mIoU, mPA, FPS, true-positive rate (TP), and inference time. UNet++, BiSeNet, HRNet, and PP-LiteSeg occupied the largest radial areas, indicative of superior overall performance relative to the other models. In contrast, SegNeXt-T and SegFormer-B1 showed noticeably smaller areas, consistent with their lower mIoU under the unified test protocol. Figure 7 illustrates the trade-off between segmentation accuracy (quantified by mIoU) and computational efficiency (quantified by FPS) as a scatter plot. UNet++ was positioned at the top of the plot, reflecting the highest segmentation accuracy (mIoU = 0.9543), although its inference speed was the lowest among the deep learning models. BiSeNet was situated in the far-right region, denoting the fastest inference speed (310.03 FPS) with competitive accuracy. HRNet and PP-LiteSeg achieved a balanced compromise between accuracy and speed, clustering in the upper-right region. SegFormer-B1 and SegNeXt-T clustered in the central-lower region, showing moderate accuracy and speed. Swin-Tiny and OCRNet showed moderate-to-low performance, while the conventional algorithms were concentrated in the lower-left quadrant, characterized by suboptimal accuracy and efficiency.

**Figure 8:**
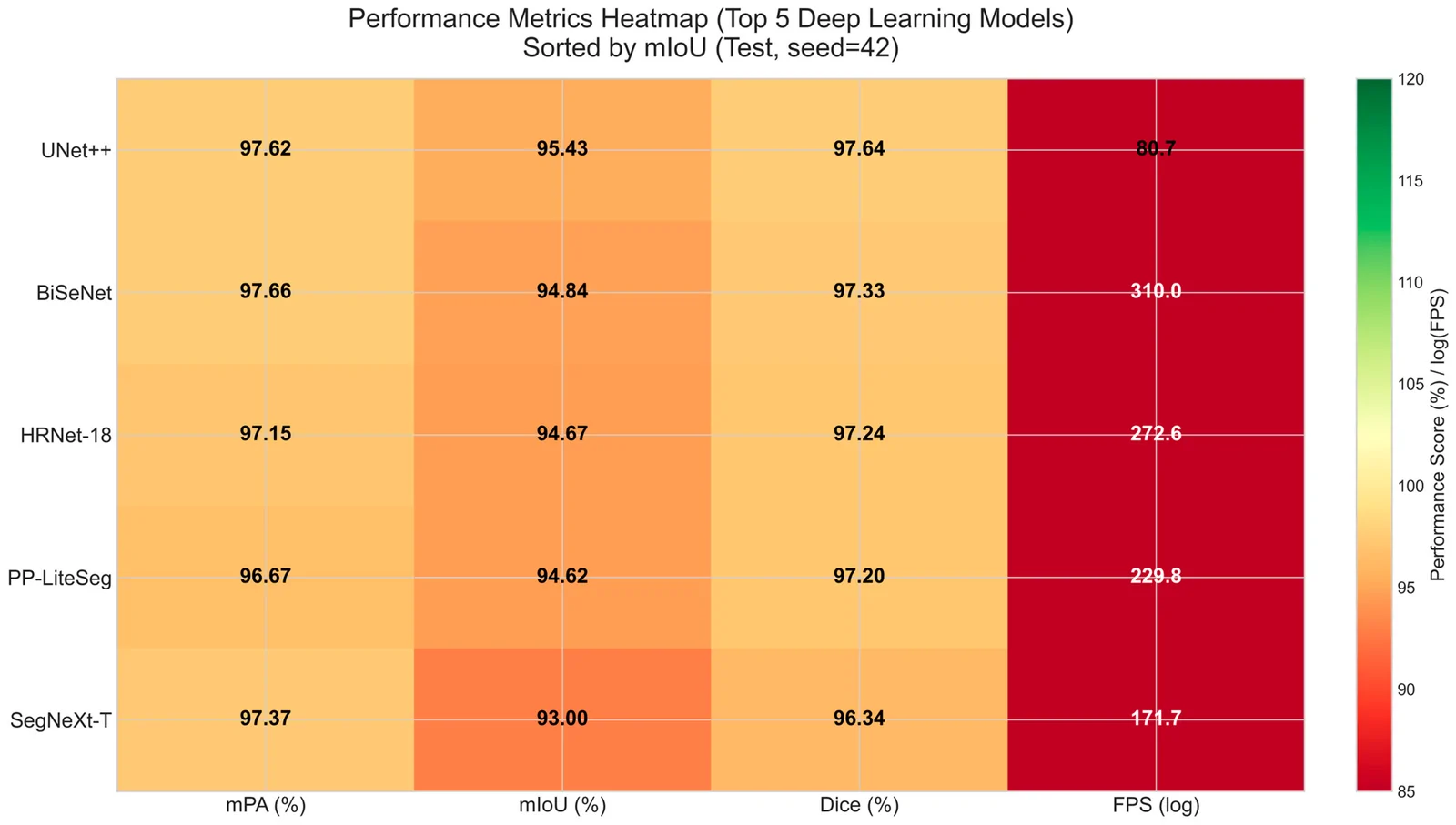
Normalized heatmap of model performance across evaluation metrics (mIoU, mPA, Dice, FPS, TP, and inverse inference time). Darker shading indicates superior performance. The top five deep learning models are shown, sorted by test mIoU (seed = 42).

**Figure 9:**
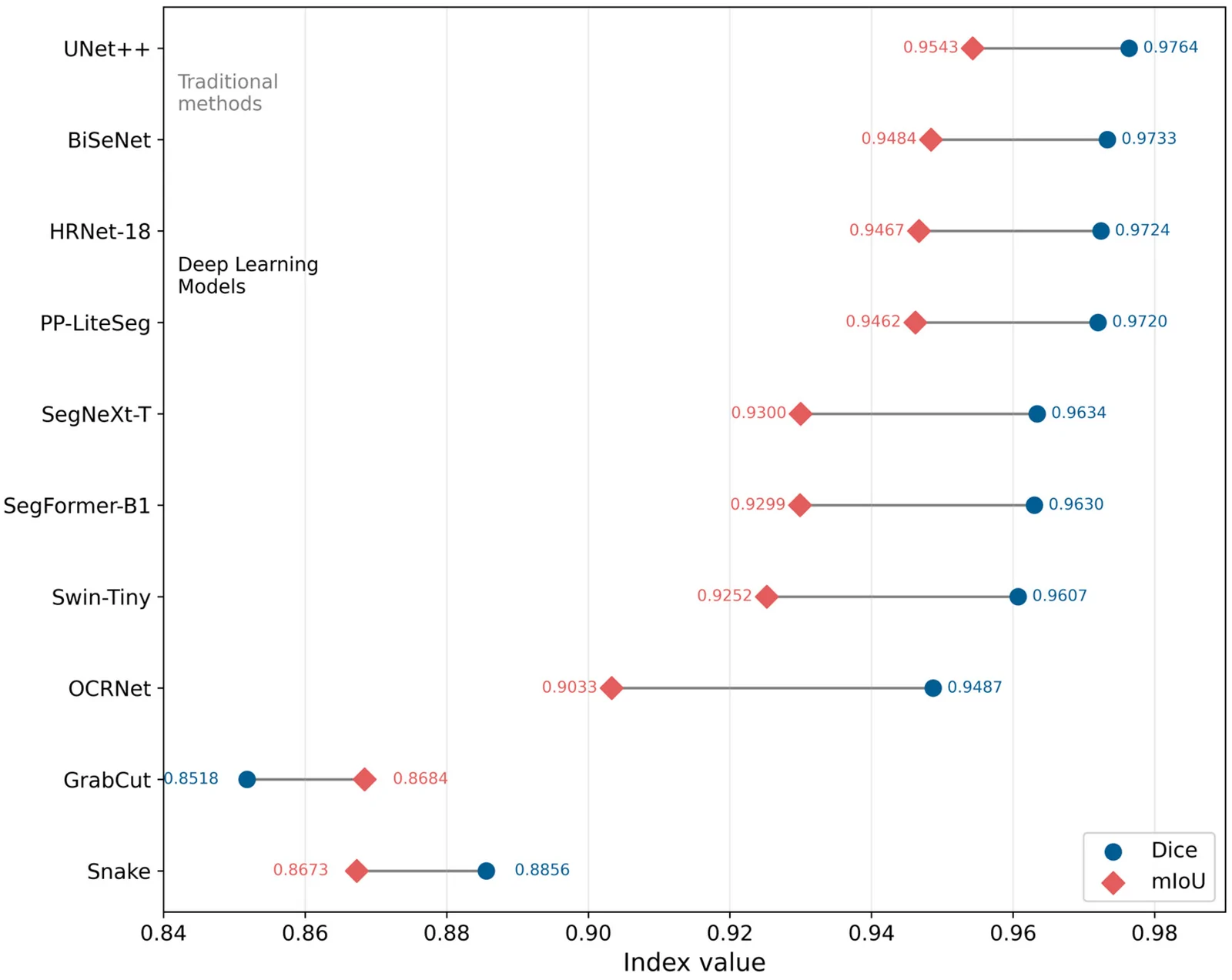
Grouped bar chart comparing Dice coefficients and mIoU values for each model.

**Figure 10:**
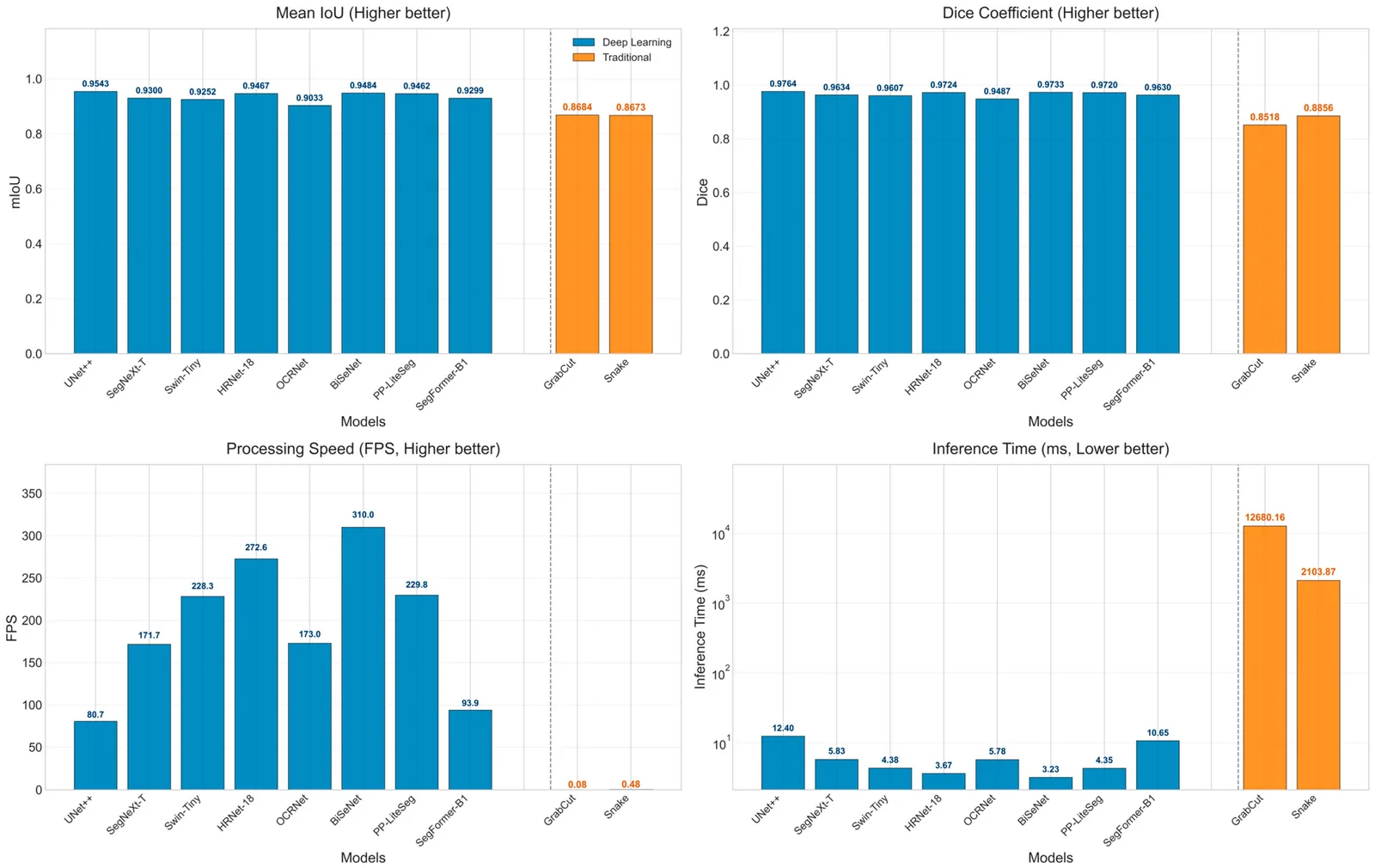
Aggregate performance comparison between conventional and deep learning algorithms (mean values) across evaluation metrics.

Figure 8 displays a normalized heatmap of the performance metrics for the top five deep learning models. UNet++ exhibited the highest normalized values for mIoU (95.43), while BiSeNet showed the highest mPA (97.66) and the fastest inference speed (310.0 FPS). HRNet and PP-LiteSeg demonstrated a more uniform distribution across all metrics. Figure 9 presents a bar chart comparing the Dice coefficient and mIoU across all models; the two metrics exhibited a highly concordant trend. UNet++ achieved the highest scores on both indicators (mIoU = 0.9543, Dice = 0.9764), followed by BiSeNet (mIoU = 0.9484, Dice = 0.9733) and HRNet (mIoU = 0.9467, Dice = 0.9724). The conventional algorithms yielded the lowest values. Figure 10 compares the mean performance of conventional and deep learning algorithms. The deep learning models demonstrated markedly superior performance on core indicators, including mIoU, mPA, and FPS, with an approximate 9% relative improvement in mIoU and more than two orders of magnitude increase in FPS compared with the traditional methods. Collectively, these findings substantiate the accuracy and efficiency advantages of lightweight deep learning architectures for tongue image segmentation.

### 4.4. Ablation Study

To validate the contribution of the UAFM and the attention mechanism in PP-LiteSeg, we conducted an ablation study on the independent test set of 150 tongue images. Three configurations were compared: (1) the full model (Baseline), (2) the model without UAFM (w/o UAFM), and (3) the model with UAFM but without the attention mechanism (w/o Attention).

As shown in Table 7, the full model achieved the best mIoU (0.9453) and Dice (0.9717), outperforming both w/o UAFM (mIoU = 0.9376, Dice = 0.9675) and w/o Attention (mIoU = 0.9382, Dice = 0.9678). Removing UAFM reduced the parameter count by approximately 48% (from 980,161 to 512,065) and improved the inference speed from 181.5 FPS to 220.9 FPS, but at the cost of a 0.0077 drop in mIoU. Removing the attention mechanism reduced the parameter count slightly (from 980,161 to 955,201) and improved the inference speed to 212.6 FPS, but reduced mIoU by 0.0071.

These results indicate that both UAFM and the attention mechanism contribute positively to segmentation accuracy, and that removing either module leads to a measurable performance drop. The simplified configurations achieve higher throughput with fewer parameters, but this efficiency gain comes at the cost of accuracy, demonstrating a clear accuracy–efficiency trade-off. This finding suggests that the full PP-LiteSeg configuration provides the best balance between segmentation accuracy and computational efficiency for tongue image segmentation.

### 4.5. Mobile Deployment Validation

**Figure 11:**
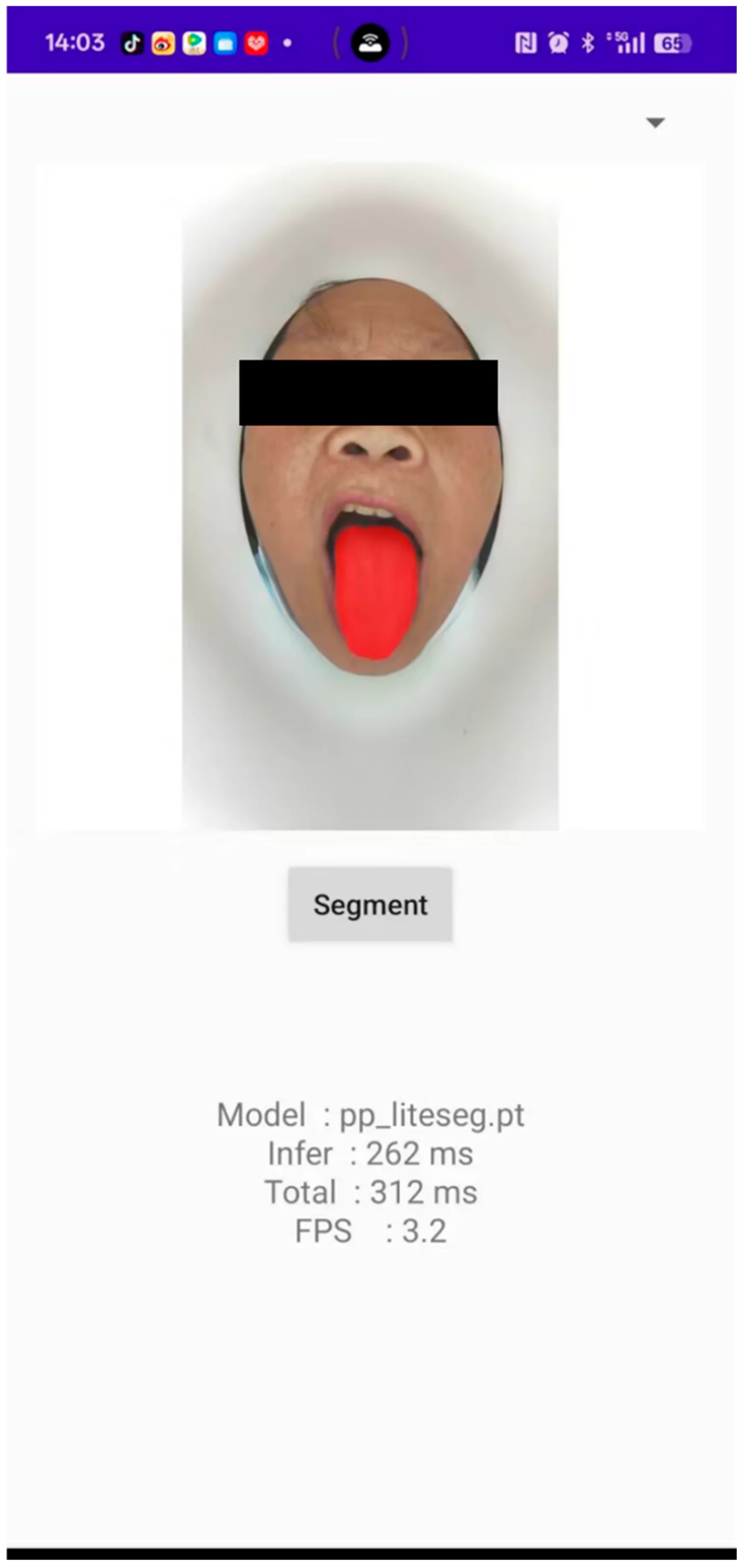
Mobile deployment validation on an OPPO Reno9 5G (Snapdragon 778G). The PP-LiteSeg model was deployed via a custom Android application, achieving 262 ms inference latency (3.2 FPS), Red indicates the predicted tongue mask.

To validate the feasibility of deploying the lightweight model on real mobile devices, we deployed the PP-LiteSeg model on three Android devices with different chipsets: HUAWEI MatePad 11.5 (Kirin T82, Huawei Technologies Co., Ltd., Shenzhen, China), Honor ANN-AN00 (Snapdragon 782G, Honor Device Co., Ltd., Shenzhen, China), and OPPO Reno9 5G (Snapdragon 778G, Guangdong OPPO Mobile Telecommunications Corp., Ltd., Dongguan, China). The model was converted to TorchScript format and deployed via a custom Android application. Table 8 summarizes the measured inference latency, total processing time (including preprocessing and postprocessing), and throughput. The inference latency ranged from 233 ms to 262 ms, the total processing time ranged from 296 ms to 317 ms, and the throughput ranged from 3.2 to 3.4 FPS. Figure 11 shows the deployment on an OPPO Reno9 5G (Snapdragon 778G), where the measured inference latency was 262 ms and the throughput was 3.2 FPS. These results demonstrate the feasibility of deploying lightweight segmentation models on portable devices with limited computational resources. Further optimization, such as INT8 quantization and hardware-specific acceleration, is expected to improve throughput in future work.

## 5. Discussion

### 5.1. Main Findings and Architectural Analysis

The pronounced superiority of deep learning models for tongue image segmentation arises from their end-to-end hierarchical feature learning capabilities. In contrast, conventional GrabCut and Snake methods depend on hand-crafted low-level features, including chromatic distributions and image gradients, which are inherently inadequate for coping with common clinical artifacts, such as indistinct lingual margins, non-uniform illumination, and complex textural patterns on the tongue surface. By contrast, deep learning architectures are capable of automatically extracting hierarchical feature representations encompassing both textural details and semantic information from large-scale datasets. In the present study, four lightweight models achieved mIoU values exceeding 0.94, with OCRNet showing the lowest performance (mIoU = 0.9033), thereby substantiating the efficacy of this methodological paradigm.

From an architectural perspective, the lightweight designs exhibited substantial performance heterogeneity on the tongue image segmentation task. UNet++ achieved the highest test mIoU (0.9543) and Dice (0.9764), demonstrating that dense skip connections and multi-scale feature fusion are particularly effective for tongue segmentation. However, its inference time (12.40 ms, 80.65 FPS) was the longest among the evaluated models, rendering it more suitable for offline or server-side analysis rather than real-time deployment. BiSeNet achieved the highest throughput (310.03 FPS) together with the highest mPA (0.9766) and a competitive mIoU (0.9484), making it the most efficient model for real-time mobile applications. HRNet maintained high-resolution representations throughout the network and achieved a competitive mIoU (0.9467) with the second-fastest inference speed (272.64 FPS), positioning it as a strong candidate for high-resolution, real-time tongue segmentation. PP-LiteSeg, which incorporates a lightweight encoder and a unified attention fusion module, achieved a competitive mIoU (0.9462) with a minimal parameter count, confirming the effectiveness of its overall architecture. SegNeXt-T and SegFormer-B1 achieved moderate mIoU values (0.9300 and 0.9299, respectively), suggesting that multi-scale convolutional attention and position-free Transformer encoding did not fully capitalize on their architectural advantages in this single-target, boundary-sensitive context. Swin-Tiny and OCRNet exhibited the lowest performance (mIoU = 0.9252 and 0.9033, respectively), indicating that window attention and object context mechanisms may require larger datasets or different adaptation strategies to reach their full potential in tongue image segmentation. Notably, recent lightweight variants of large-scale models, including TongueLiteSAM, MEATSAM, and TongueSAM_Lite, have achieved considerable model compression while preserving zero-shot capabilities through knowledge distillation and the substitution of lightweight encoders. Nevertheless, the present findings indicate that these state-of-the-art models have not yet exhibited systematic superiority over mainstream lightweight CNN and Transformer architectures on authentic clinical tongue image datasets, and their readiness for practical deployment warrants further investigation.

### 5.2. Model Selection and Clinical Implications

Relative to conventional approaches, the deep learning models evaluated in the present study achieved an approximate 9% relative improvement in mIoU and increased inference speed by more than two orders of magnitude. These findings are consistent with prior reports indicating that conventional methods exhibit limited robustness and computational inefficiency in complex tongue image scenarios. The principal advantages of conventional algorithms lie in their independence from training data and their mechanistic transparency, rendering them appropriate as baseline references. Nevertheless, they fail to satisfy real-time requirements in practical deployments. The comparative analyses in the present study, conducted on a unified hardware platform and evaluation protocol, further quantified the performance disparity between conventional and deep learning approaches and substantiated the feasibility of lightweight architectures for tongue image segmentation.

Integrating accuracy and efficiency metrics, the present findings offer objective criteria for model selection across diverse clinical deployment scenarios. Among the evaluated models, UNet++ achieved the highest test mIoU (0.9543), while BiSeNet achieved the highest throughput (310.03 FPS) and the highest mPA (0.9766). PP-LiteSeg (mIoU = 0.9462, 229.75 FPS) and HRNet (mIoU = 0.9467, 272.64 FPS) provided favorable balances between accuracy and efficiency. For scenarios requiring stringent real-time performance, including mobile tongue-diagnosis applications, portable devices, and continuous video stream processing, BiSeNet is the most suitable candidate, owing to its highest throughput (310.03 FPS) and competitive accuracy (mIoU = 0.9484). For offline high-precision diagnostic tasks, scientific research analyses, or applications necessitating precise extraction of edge features, including cracks and tooth marks, UNet++ exhibited the highest segmentation accuracy (mIoU = 0.9543), although its inference speed (80.65 FPS) is more suitable for server-side deployment. For routine outpatient auxiliary diagnosis or batch processing of health examination data, HRNet and PP-LiteSeg offered balanced trade-offs between accuracy and efficiency. Collectively, these findings establish a systematic framework for model selection in resource-constrained and mobile deployment scenarios, addressing a notable gap in the existing literature.

In the broader context of advancing objective tongue diagnosis, high-quality segmentation constitutes the prerequisite for subsequent quantitative analyses, including chromatic assessment of the tongue body, tongue coating classification, and detection of morphological features such as fissures and tooth marks. Accurate and complete extraction of the lingual region is essential to ensure the reliability of downstream analyses. The present study demonstrated that lightweight deep learning architectures can achieve high-precision segmentation even in resource-constrained environments, exemplified by the RTX 3050 Ti platform and the deployment on three Android devices (HUAWEI MatePad 11.5, Honor ANN-AN00, and OPPO Reno9 5G), thereby substantiating the feasibility of deploying such models on mobile terminals or portable diagnostic devices. This advancement is anticipated to progressively transform traditional Chinese medicine tongue diagnosis from reliance on physicians’ subjective experience toward a measurable, reproducible, and statistically grounded objective evaluation framework, thereby facilitating the standardization and broader dissemination of traditional Chinese medicine diagnostic methodologies.

### 5.3. Failure Case Analysis Under Challenging Clinical Conditions

**Figure 12:**
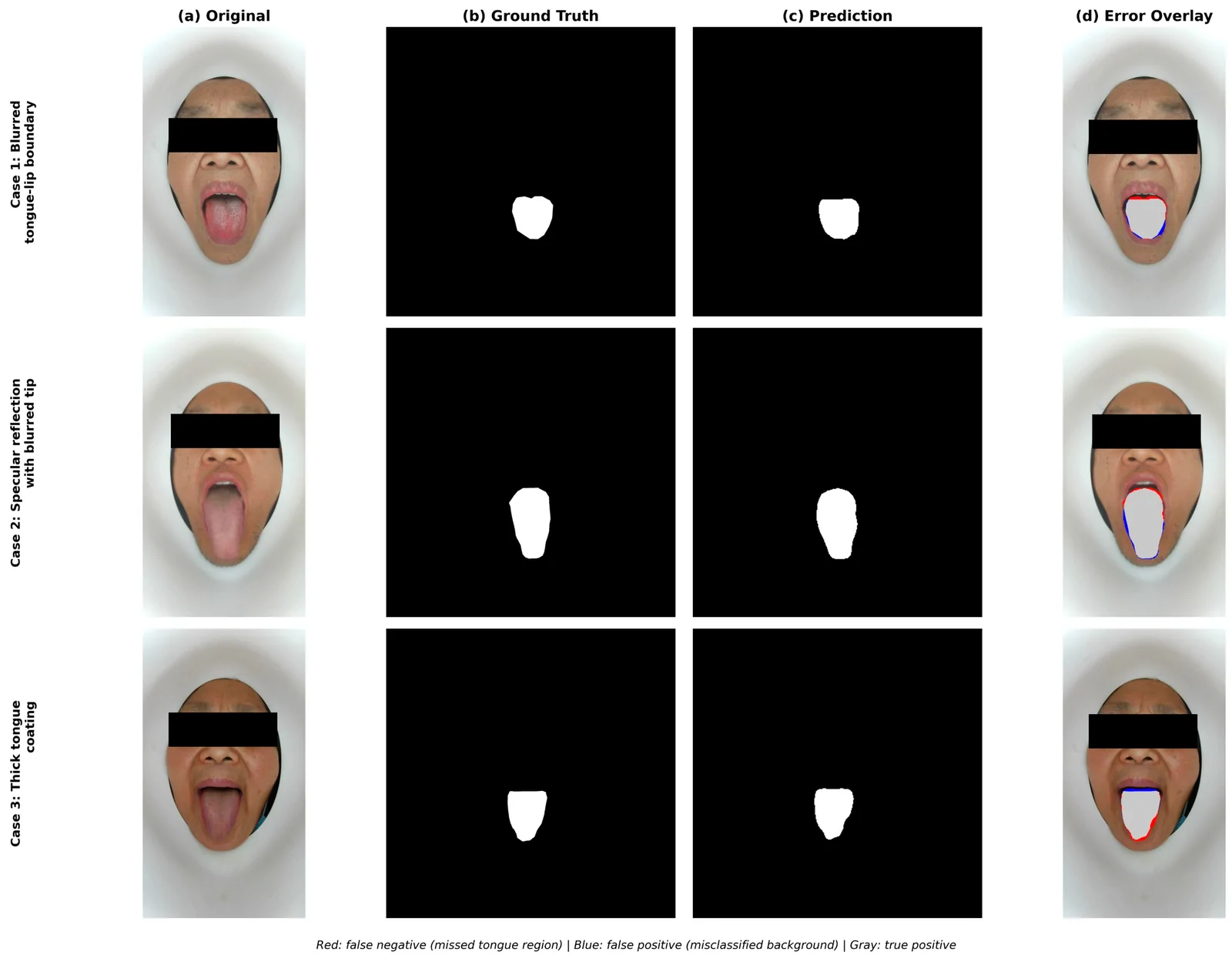
Representative failure cases on the independent test set. Columns: (**a**) original, (**b**) ground truth, (**c**) prediction, (**d**) error overlay. Cases 1–3: blurred tongue–lip boundary, specular reflection, and thick tongue coating. Red: FN; blue: FP.

To evaluate the robustness of the proposed method under challenging clinical conditions, we conducted a qualitative analysis of three representative failure cases on the independent test set (Figure 12).

Case 1: Blurred tongue–lip boundary. In this case, the tongue tip is in close contact with the lower lip, and their chromatic characteristics are highly similar. The model misclassified part of the lower lip as tongue (blue region, FP) and missed the tongue base near the oral cavity (red region, FN). This indicates that boundary ambiguity between the tongue and adjacent tissues remains a major challenge.

Case 2: Specular reflection with blurred tip. Strong specular highlights appear on the tongue surface, causing severe chromatic distortion. The model misclassified the highlighted region as background (red region, FN). This suggests that the model relies heavily on chromatic features and is sensitive to illumination variations.

Case 3: Thick tongue coating with partial occlusion. The tongue surface is covered by a thick coating, and the tongue base is partially occluded by oral shadow. The model missed the coating region and the tongue base (red region, FN). This indicates that thick coatings and occluded boundaries are still difficult for the current model.

These failure cases indicate that the current model is still limited under blurred boundaries, non-uniform illumination, and thick coatings. Future work will address these issues by incorporating illumination normalization, boundary-aware refinement, and data augmentation with more diverse coating conditions.

### 5.4. Limitations and Future Work

The present study is subject to several limitations. First, although the mobile deployment validation was performed on three Android devices with different chipsets (HUAWEI MatePad 11.5, Honor ANN-AN00, and OPPO Reno9 5G) using the PP-LiteSeg model, the inference latency ranged from 233 to 262 ms and the throughput from 3.2 to 3.4 FPS. Memory usage and power consumption were not measured due to hardware limitations, and a comprehensive evaluation across a wider range of devices and models remains to be conducted. Second, the study was restricted to static tongue image segmentation and did not account for dynamic lingual movements, such as deformations associated with tongue protrusion or continuous segmentation in video streams. This limitation constrains its applicability in real-world dynamic clinical scenarios. Third, model interpretability and clinical acceptance remain to be assessed. Fourth, although we performed multi-seed training (3 random seeds: 42, 123, 2024) and reported 95% confidence intervals for the four top-performing models (UNet++, BiSeNet, PP-LiteSeg, and HRNet), the remaining models (SegNeXt-T, SegFormer-B1, Swin-Tiny, and OCRNet) were trained with a single seed due to limited computational resources. Consequently, the small performance differences among the top-performing models should be interpreted with caution, and the relative ranking of the remaining models may not be statistically robust.

Future investigations will address these limitations in the following directions. First, the mobile deployment will be extended to a wider range of devices (e.g., flagship and mid-range smartphones) and multiple models, with measurements of memory usage and power consumption. Second, temporal information modeling and dynamic tongue image analysis will be incorporated to extend the current framework from static images to continuous video streams. Third, model interpretability techniques will be explored, and prospective trials will be conducted in collaboration with clinical departments to collect feedback from physicians and patients, thereby further validating model stability and acceptability in authentic clinical settings. Fourth, multi-seed training will be extended to all eight models to estimate confidence intervals and conduct statistical significance tests across the full model set. In addition, lightweight optimization techniques such as INT8 quantization and knowledge distillation will be investigated, and a streamlined tongue diagnosis application prototype will be developed to establish a closed-loop pipeline from image acquisition to segmentation output. Dataset scale and diversity will also be augmented, and multi-center validation will be performed to facilitate the translation of these findings from laboratory research to clinical practice.

## 6. Conclusions

TCM tongue diagnosis is non-invasive and inherently holistic; nevertheless, its clinical application relies substantially on physicians’ subjective expertise, thereby limiting objectivity and standardization. As the foundational step toward the objectification of tongue diagnosis, tongue image segmentation directly determines the reliability of subsequent feature extraction.

This study systematically compared the performance of two conventional segmentation algorithms and eight lightweight deep learning models under a standardized protocol. Experimental results demonstrated that lightweight deep learning models consistently outperformed conventional approaches in both accuracy and computational efficiency, with mean Intersection over Union (mIoU) improving by approximately 9% and inference speed increasing by more than two orders of magnitude.

The evaluated architectures represented mainstream technical paradigms, including encoder–decoder structures, high-resolution representation preservation, dual-branch real-time networks, lightweight Transformers, and attention-based mechanisms.

Among the evaluated models, UNet++ achieved the highest test mIoU (0.9543) and Dice (0.9764), and paired bootstrap analysis confirmed that its advantage over BiSeNet, PP-LiteSeg, and HRNet was statistically significant. BiSeNet achieved the highest throughput (310.03 FPS) together with the highest mPA (0.9766), making it the most efficient model for real-time mobile applications. HRNet (mIoU = 0.9467, 272.64 FPS) and PP-LiteSeg (mIoU = 0.9462, 229.75 FPS) provided a favorable balance between accuracy and efficiency, rendering them suitable for real-time mobile-based tongue diagnosis. Overall, these findings establish a technical foundation for the intelligent modernization and standardization of TCM tongue diagnosis, bridging the gap between algorithmic development and clinical practice.

## Figures and Tables

**Table 1:** Comparison of lightweight adaptations for eight deep learning models.

Model	Year	Core Characteristics of the Original Architecture	Lightweight Adaptation in This Study	Principal Simplifications and Modifications
UNet++	2018	Encoder–decoder architecture with nested dense skip connections and deep supervision	Nested U-shaped topology with VGGBlocks (channels: 32, 64, 128, 256, 512) and a single output head	Removal of deep supervision; reduction in channel complexity; replacement of standard convolutions with VGGBlocks
SegNeXt-T	2022	Hierarchical convolution with MSCA-based parallel large-kernel attention	Four-stage architecture (channels: 32, 64, 128, 256); serial MSCA (5 × 5 DWConv, 1 × 7 and 7 × 1 convolutions); lightweight decoder with upsampling and concatenation	Reconfiguration of MSCA from parallel to serial; decoder lightweighting
Swin-T	2021	Hierarchical Transformer with window/shifted-window self-attention and relative position bias	Purely convolutional design: four-stage convolutional patch embedding; 1 × 1 group convolution emulating multi-head attention; stepwise convolutional downsampling; omission of positional bias	Replacement of window/shifted-window attention with fully convolutional operations; significant inference acceleration
HRNet	2019	Parallel multi-resolution branches with repeated cross-resolution fusion	LightHRNet18: serial single-branch architecture maintaining 1/4 resolution post-stem, using BasicBlocks (channels: 16, 32, 64, 128)	Simplification to a serial architecture retaining high-resolution representations; elimination of multi-branch fusion
OCRNet	2020	Backbone (typically HRNet) with object context representation (aggregation of multi-region soft segmentation)	MobileNetV2 backbone; simplified OCRBlock: global average pooling (GAP) and 1 × 1 convolution for object representation, followed by point-wise multiplication with pixel representation	Backbone replacement with MobileNetV2; simplification of object representation; removal of multi-region soft segmentation
BiSeNet	2018	Dual-branch architecture: spatial path (high resolution), context path (rapid downsampling), and fusion module	Detail branch (three-stage convolution, 1/4 scale), semantic branch (stem downsampling to 1/32), and element-wise addition for fusion	Simplification of integration without bidirectional guidance; lightweight semantic branch
PP-LiteSeg	2022	STDC lightweight backbone with unified attention fusion module (AFM) and lightweight FPN	Custom encoder (stride convolution and depthwise separable convolutions; channels: 32, 64, 128, 256); UAFM incorporating channel and spatial attention; width multiplier of 0.5 (T version)	Omission of STDC backbone; retention of UAFM core mechanism
SegFormer-B1	2021	MiT hierarchical Transformer encoder with MLP-based decoder; omission of positional encoding	MixVisionTransformer encoder with SegFormer head; four-stage overlapping PatchEmbed; attention with spatial reduction ratios of 8, 4, 2, and 1; decoder projecting to 256 dimensions with upsampling and concatenation	Preservation of original framework; adherence to B1 configuration

**Table 2:** Training hyperparameters and configurations of the deep learning models.

Model Name	Batch Size	Optimizer	Initial Learning Rate	Loss Function	Other Settings
UNet++	4	Adam	1 × 10^−3^	BCEWithLogitsLoss	TensorBoard records the training process
SegNeXt-T	8	AdamW	1 × 10^−3^	BCEWithLogitsLoss	The training duration is relatively long.
Swin-Tiny	8	Adam	1 × 10^−3^	BCEWithLogitsLoss	The number of parameters of the statistical model in the code
HRNet	8	AdamW	1 × 10^−3^	BCEWithLogitsLoss	The batch size can be adjusted when the video memory is insufficient.
OCRNet	8	AdamW	1 × 10^−3^	BCEWithLogitsLoss	The training process is quite standardized.
BiSeNet	8	AdamW	1 × 10^−3^	BCEWithLogitsLoss	Adopt the standard supervision training process
PP-LiteSeg	8	AdamW	1 × 10^−3^	DiceLoss + BCEWithLogitsLoss	Set up adaptive adjustment of the learning rate
SegFormer-B1	8	AdamW	1 × 10^−4^	BCEWithLogitsLoss	Lower learning rate for stable training.

**Table 3:** Comparison of two conventional image segmentation algorithms for tongue image segmentation.

Model Name	Inference Time (ms)	FPS	mIoU	mPA
Grabcut	12,680.16	0.08	0.8684	0.8803
Snake	2103.87	0.48	0.8673	0.8831

**Table 4:** Comparison of eight deep learning models for tongue image segmentation. Best results are shown in bold; second-best results are underlined.

Model Name	Inference Time (ms)	FPS	mIoU	mPA
**UNet++**	12.40	80.65	**0.9543**	0.9762
**BiSeNet**	**3.23**	**310.03**	0.9484	**0.9766**
**PP-LiteSeg**	4.35	229.75	0.9462	0.9667
**HRNet**	3.67	272.64	0.9467	0.9715
**SegNeXt-T**	5.83	171.66	0.9300	0.9737
**SegFormer-B1**	10.65	93.92	0.9299	0.9584
**Swin-Tiny**	4.38	228.28	0.9252	0.9619
**OCRNet**	5.78	172.96	0.9033	0.9525

**Table 5:** For the four top-performing models, we report the mean ± 95% confidence interval over 3 random seeds (42, 123, 2024). CI computed via t-distribution with df = 2.

Model	Test Dice (Mean ± 95% CI)	Test mIoU (Mean ± 95% CI)
UNet++	0.9759 ± 0.0011	0.9533 ± 0.0022
BiSeNet	0.9730 ± 0.0017	0.9479 ± 0.0032
HRNet	0.9727 ± 0.0017	0.9475 ± 0.0032
PP-LiteSeg	0.9716 ± 0.0028	0.9453 ± 0.0052

**Table 6:** Paired bootstrap (10,000 resamples) on per-image Dice scores. All 95% CIs lie entirely above zero, indicating statistically significant differences.

Comparison	ΔDice	95% CI
UNet++ vs. BiSeNet	+0.0031	[+0.0023, +0.0038]
UNet++ vs. PP-LiteSeg	+0.0043	[+0.0031, +0.0056]
UNet++ vs. HRNet	+0.0040	[+0.0030, +0.0052]

**Table 7:** Ablation study on the PP-LiteSeg model, evaluated on the independent test set (150 images). Baseline includes both UAFM and attention modules. w/o UAFM removes the unified attention fusion module; w/o Attention removes the attention mechanism while retaining UAFM. Best results are shown in bold; second-best results are underlined.

Configuration	UAFM	Attention	Params	mIoU	Dice	FPS
Baseline	Yes	Yes	980,161	**0.9453**	**0.9717**	181.5
w/o UAFM	No	No	512,065	0.9376	0.9675	**220.9**
w/o Attention	Yes	No	955,201	0.9382	0.9678	212.6

**Table 8:** Mobile deployment validation across three Android devices.

Device	Chipset	OS	Inference (ms)	Total (ms)	FPS
HUAWEI MatePad 11.5	Kirin T82	HarmonyOS 6.1	233	296	3.4
Honor ANN-AN00	Snapdragon 782G	MagicOS 9.0	233	317	3.2
OPPO Reno9 5G	Snapdragon 778G	ColorOS 15.0	262	312	3.2

## Data Availability

The data presented in this study are available on request from the corresponding author due to privacy and ethical restrictions.

## Supplementary Materials

The following supporting information can be downloaded at: https://www.mdpi.com/article/10.3390/bioengineering13101201/s1, Figure S1: Training convergence curves; Figure S2: Generalization check; Table S1: Training, validation, and test performance.
