# Supplementary material for: Research and Application of Tongue Image Segmentation Method Based on Lightweight Deep Learning
Source: Bioengineering (Basel). 2026 Oct 15;13(10):1201. doi: 10.3390/bioengineering13101201 (PMC13701977; doi:10.3390/bioengineering13101201)
Supplement: Supplementary file 1 [file bioengineering-13-01201-s001.zip › bioengineering-4550790-supplementary.pdf]

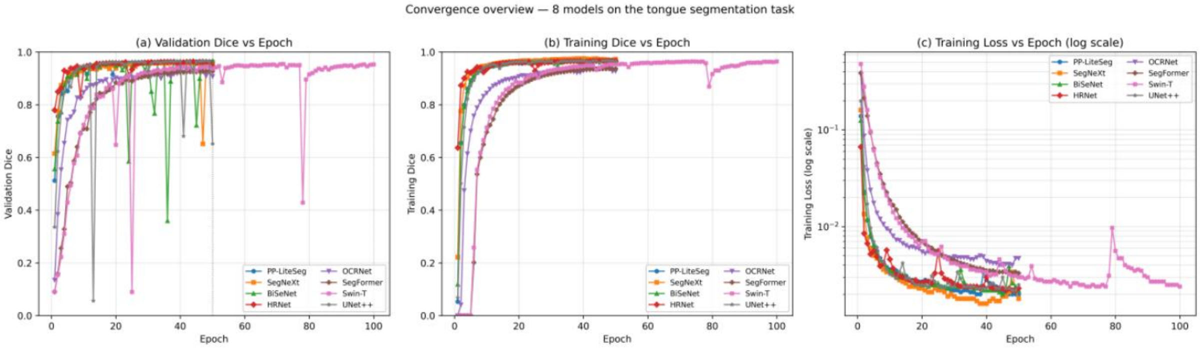

Supplementary Figure S1. Figure S1. Convergence overview of eight segmentation models on the tongue segmentation task (seed 42). (a) Validation Dice, (b) Training Dice, and (c) Training loss (log scale) as functions of epoch. Vertical dotted lines indicate the last training epoch of each model (50 epochs for most models; Swin-T was trained for 100 epochs due to its slower convergence). All models converge within 20–30 epochs. Local dips in the validation Dice curves (e.g., UNet++, HRNet, Swin-T) correspond to single-epoch fluctuations in BatchNorm statistics and do not indicate training divergence, as confirmed by the stable training curves in panels (b) and (c).

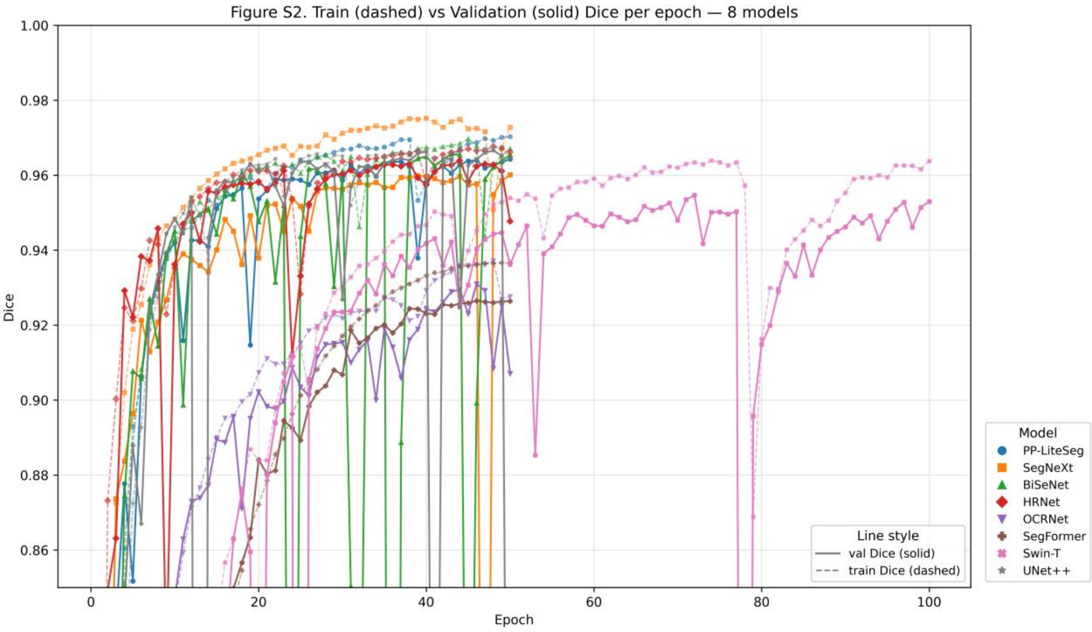

Supplementary Figure S2. Generalization check: per-epoch training Dice (dashed) and validation Dice (solid) for eight segmentation models. The train–validation gap remains small (<0.05 for most models) throughout training, indicating no significant overfitting. Curves end at each model's final epoch (50 epochs for most models; Swin-T: 100 epochs). The narrow gap across all models demonstrates that the models generalize well from training to validation data.

Table S1. Comparison of training, validation, and test performance of eight segmentation models (SegNeXt-T, BiSeNet, PP-LiteSeg, SegFormer-B1, UNet++, HRNet-18, Swin-Tiny, and OCRNet). All experiments use the same training/validation/test splits of 700/150/150 images. For each model, we report Dice and mIoU on all three splits, computed with the same evaluation script and a fixed threshold of 0.5. The small gap between training and validation performance indicates that no severe overfitting occurs, and the consistency between validation and test performance confirms that model selection based on the validation set generalizes well to unseen data. Best test performance is shown in bold.

Model	Train Dice	Val Dice	Test Dice	Train mIoU	Val mIoU	Test mIoU
UNet++	0.9774	0.9748	0.9764	0.9563	0.9517	0.9543
BiSeNet	0.9770	0.9716	0.9733	0.9556	0.9455	0.9484
PP-LiteSeg	0.9795	0.9696	0.9720	0.9603	0.9421	0.9462
HRNet-18	0.9767	0.9699	0.9724	0.9550	0.9426	0.9467
SegNeXt-T	0.9762	0.9602	0.9634	0.9550	0.9248	0.9300
SegFormer-B1	0.9782	0.9599	0.9630	0.9577	0.9248	0.9299
Swin-Tiny	0.9745	0.9586	0.9607	0.9508	0.9216	0.9252
OCRNet	0.9572	0.9422	0.9487	0.9189	0.8925	0.9033
